# Neuralized family member NEURL1 is a ubiquitin ligase for the cGMP-specific phosphodiesterase 9A

**DOI:** 10.1038/s41598-019-43069-x

**Published:** 2019-05-08

**Authors:** Kati Taal, Jürgen Tuvikene, Grete Rullinkov, Marko Piirsoo, Mari Sepp, Toomas Neuman, Richard Tamme, Tõnis Timmusk

**Affiliations:** 1 Department of Chemistry and Biotechnology, Tallinn University of Technology, Akadeemia tee 15, 12618 Tallinn, Estonia; 2 Protobios LLC, 12618 Tallinn, Estonia; 3 Present Address: Institute of Technology, University of Tartu, Nooruse 1, 50411 Tartu, Estonia; 4 Present Address: Center for Molecular Biology of Heidelberg University (ZMBH), DKFZ-ZMBH Alliance, D-69120 Heidelberg, Germany

**Keywords:** Ubiquitylation, Ubiquitin ligases, Molecular neuroscience

## Abstract

Neuralized functions as a positive regulator of the Notch pathway by promoting ubiquitination of Notch ligands via its E3 ligase activity, resulting in their efficient endocytosis and signaling. Using a yeast two-hybrid screen, we have identified a cGMP-hydrolysing phosphodiesterase, PDE9A, as a novel interactor and substrate of Neuralized E3 ubiquitin protein ligase 1 (NEURL1). We confirmed this interaction with co-immunoprecipitation experiments and show that both Neuralized Homology Repeat domains of NEURL1 can interact with PDE9A. We also demonstrate that NEURL1 can promote polyubiquitination of PDE9A that leads to its proteasome-mediated degradation mainly via lysine residue K27 of ubiquitin. Our results suggest that NEURL1 acts as a novel regulator of protein levels of PDE9A.

## Introduction

*Neuralized* (*Neur*) was originally identified as a neurogenic gene in *Drosophila* whose loss-of-function mutations cause neural hyperplasia at the expense of epidermis^1^. Neur protein is localised to the cytoplasmic side of the cell membrane and functions as an E3 ubiquitin ligase for Delta and other Notch ligands^2^. Ubiquitination of Delta is required for its endocytosis that facilitates, via multiple proteolytic processing acts, translocation of the Notch intracellular domain into the nucleus and activation of neurogenic *Hes* genes together with transcriptional coactivators Suppressor-of-Hairless and Mastermind^3^. Notch signalling is important for numerous developmental, physiological and pathological processes^3^. Its misregulation has been implicated in a large number of diseases and conditions, thus making it an attractive therapeutic target^4^.

*Drosophila* Neur (dNeur) protein has two Neuralized Homology Repeat (NHR) domains and a C-terminal C3HC4 RING Zn-finger (RING) domain^5^. In mammals, there are at least four proteins with NHR domains: NEURL1 (or NEURL, NEURL1A, NEUR1) and NEURL1B (or NEUR2) both have two NHR domains and a C-terminal RING domain, whereas NEURL2 (or Ozz-E3) and NEURL3 (or LINCR) have only one NHR domain and a C-terminal SOCS (suppressor of cytokines signalling) or RING domain, respectively^6–9^. Mammalian NEURL1 and NEURL1B proteins interact with and ubiquitinate Notch ligands Delta-like1, Delta-like4 and Jagged1^7,10,11^.

In both flies and mammals, Neur has been shown also to act as an ubiquitin ligase for substrates other than Notch ligands. In flies, Neur promotes ubiquitination of the Crumbs complex protein Stardust (Std) to down-regulate the levels of Std isoforms possessing the Neuralized Binding Motif. Therefore, in this context Neur is involved in remodelling of epithelium in the posterior midgut, thus promoting the trans-epithelial migration of the primordial germ cells in early fly embryos^12^. In mice, NEURL1-mediated monoubiquitination of CPEB3 (cytoplasmic polyadenylation element binding protein 3) leads to increased translation levels of GRIA1 and GRIA2 (glutamate ionotropic receptor AMPA – α-Amino-3-hydroxy-5-methyl-4-isoxazolepropionic acid – type subunits 1 and 2 or GluA1 and GluA2) and concomitant enhancement of synaptic plasticity and spatial memory^13^.

Subcellular localisation of dNeur and NEURL1 has not been unequivocally established. While we have shown that, in Neuro2A cells, overexpressed NEURL1 is predominantly localised either in the nucleus or in the cytoplasm^14^, another study using a different cell line found that overexpressed NEURL1 localises in the cell membrane in an N-myristoylation-dependent manner^10^. A third group found that dNeur can associate with the nuclear envelope and becomes trapped in the nucleus following inhibition of nuclear export and Delta function^15^.

Interestingly, *NEURL1* mRNA is targeted to the dendrites of the dendate gyrus of the rat adult hippocampal formation^14^ via its 3′ untranslated region^16^, implying that it may be subject to activity-dependent translational regulation and thus also have a function in adult dendrites of the dendate gyrus.

Genetic studies have suggested that, in contrast to *dNeur*, *NEURL1* and *NEURL1B* are not required for Notch signalling in mice. Two groups have independently found that while inactivation of *NEURL1* did not result in Notch-related or other developmental defects, the adult *NEURL1* knock-out phenotypes that they described were different^17,18^. One group found that male and female mice lacking *NEURL1* are sterile and have defective mammary gland differentiation, respectively^17^, while another group found these mice exhibiting hypersensitivity to ethanol and defective olfactory discrimination^18^. A third group showed that a compound knockout of *NEURL1* and *NEURL1B* does not have a Notch-like phenotype^7^. Subsequent studies have revealed that another RING-domain E3 ubiquitin ligase, Mind bomb-1, ubiquitinates Notch ligands in vertebrates *in vivo*^19^.

Ubiquitination, while initially identified as a mechanism for targeting proteins for proteasome-mediated destruction, has been shown to also function non-proteolytically to regulate endocytosis, protein trafficking, DNA repair, etc.^20^. Ubiquitination begins with the E1 enzymes (two in mammals) activating ubiquitin using ATP and then transferring it to the E2 ubiquitin-conjugating enzymes (several dozens in mammals). Subsequently, substrate-specific E3 ubiquitin ligases (several hundred in mammals) facilitate the transfer of activated ubiquitin from the E2 to designated target proteins either directly or indirectly. Importantly, a number of E3 ligases can have multiple substrates with unrelated functions^20^.

The fact that NEURL1 and NEURL1B are not required for Notch signalling suggests that they function outside of the Delta-Notch pathway. Also, both NEURL1 and NEURL1B presumably should, in common with other E3 ligases, have a number of additional substrates other than the currently known ones. Thus, a fuller understanding of NEURL proteins’ function requires identification of their putative substrates and non-substrate interactors. Here, we identify phosphodiesterase 9A (PDE9A) as a putative substrate for NEURL1 ubiquitin ligase activity. Cyclic nucleotide phosphodiesterases specifically hydrolyse either cAMP or cGMP or both cAMP/cGMP (dual-specific phosphodiesterases), crucial secondary messenger molecules important in various physiological and pathological processes^21^. PDE9A has the highest affinity for cGMP of any phosphodiesterases known^22^ and is also the cGMP-specific phosphodiesterase with the highest expression levels in the brain^23^. We show that the interaction between NEURL1 and PDE9A is mediated by both NHR domains of NEURL and that this interaction leads to ubiquitination and proteasomal degradation of PDE9A.

## Results

### NEURL1 and NEURL1B proteins interact with PDE9A

**Figure 1:**
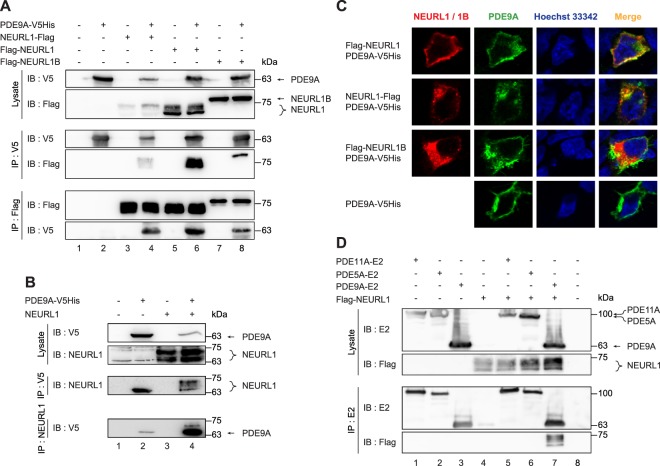
NEURL1 and NEURL1B interact with PDE9A. (**A**) PDE9A-V5His was expressed alone or together with either NEURL1-Flag, Flag-NEURL1 or Flag-NEURL1B in HEK293-FT cells. (**B**) PDE9A-V5His was expressed alone or together with untagged NEURL1 in HEK293-FT cells. In A and B the proteins were immunoprecipitated and immunoblotted using the indicated antibodies (untagged NEURL1 was detected with a custom-raised polyclonal NEURL1 antibody). (**C**) PDE9A-V5His and Flag-NEURL1 or NEURL-Flag or Flag-NEURL1B were co-expressed in HEK293 cells and their localisation was visualised by indirect immunofluorescence using respective tag-specific antibodies (co-localisation of green and red signals results in yellow additive colour in the Merge column). Nuclei were visualised using Hoechst 33342 (blue). (**D**) Flag-NEURL1 and E2-tagged PDE5A, PDE9A and PDE11A were expressed alone or in combination in HEK293-FT cells. Cell lysates were immunoprecipitated and immunoblotted with the indicated antibodies. IB, immunoblot; IP, immunoprecipitation.

To identify NEURL1-interacting proteins, a yeast two-hybrid screen was used. Screening of adult rat brain cDNA library with full-length NEURL1 as a bait resulted in total 47 positive clones. Three of these clones corresponded to PDE9A, a member of the cyclic nucleotide phosphodiesterase family, as one of the most potent interactors. To verify this interaction, we performed co-immunoprecipitation and co-localisation experiments. We also included human NEURL1B into this analysis, given its functional and sequence similarity to NEURL1. We expressed either N- or C-terminally Flag-tagged NEURL1 (to minimise tag-specific effects) or N-terminally Flag-tagged NEURL1B alone and in combination with V5His-tagged PDE9A in HEK293-FT cells, performed immunoprecipitations using Flag or V5 antibodies and analysed the immunoprecipitates by immunoblotting. We detected PDE9A in both NEURL1 and NEURL1B immunoprecipitates and both NEURLl and NEURL1B in PDE9A immunoprecipitates (Fig. 1A, lanes 4, 6 and 8), suggesting that NEURLl and NEURL1B interact with PDE9A.

*PDE9A* expression pattern in rodents overlaps with those of *NEURL1* and *NEURL1B*, for example in adult cortical and hippocampal neurons^11,14,23^, suggesting that PDE9A may interact with NEURL1 and NEURL1B *in vivo*. A PCR-based analysis revealed that both *NEURL1* and *PDE9A* are co-expressed in HEK293 cells (Supplementary Fig. 1). This suggested that it should be possible to detect an endogenous interaction between these two proteins using this cell line. However, when testing two commercially available NEURL1 antibodies, we found that neither of these could recognise endogenous NEURL1 (Supplementary Fig. 2A,B). Next we generated custom-raised polyclonal NEURL1 antibodies that showed superior specificity and sensitivity compared to the commercial NEURL1 antibodies (Supplementary Fig. 2C). We used these antibodies to determine whether endogenous or overexpressed untagged NEURL1 and V5His-tagged PDE9A interact with each other in co-immunoprecipitation experiments. To this end, we expressed untagged NEURL1 or V5His-tagged PDE9A in HEK293-FT cells and performed immunoprecipitations using NEURL1 antibodies. In a subsequent immunoblot analysis with NEURL1 antibodies, both endogenous and exogenous NEURL1 could be detected in immunoprecipitates where NEURL1 antibodies had been used (Fig. 1B, lanes 1–4 on the upper IP panel). The finding that we also detected overexpressed PDE9A in these immunoprecipitates (Fig. 1B, lanes 2 and 4 on the lower IP panel) suggests that both endogenous and overexpressed untagged NEURL1 can interact with overexpressed PDE9A. Collectively, the co-immunoprecipitation experiments suggest that both NEURL1 and NEURL1B bind PDE9A.

The observation that overexpressed NEURLl and NEURL1B interact with PDE9A implies that these proteins should have an overlapping subcellular distribution. Thus, we next analysed the intracellular distribution of NEURL1, NEURL1B and PDE9A. We used two different NEURL1 constructs, encoding Flag-NEURL1 or NEURL1-Flag (with an N- or C-terminal Flag tag, respectively), to ascertain whether the presence of the potential myristoylation signal in the N-terminus of NEURL1 (intact in NEURL1-Flag only) affects its subcellular localisation. We performed immunofluorescence analysis using HEK293 cells co-expressing PDE9A-V5-His together with Flag-NEURL1, NEURL1-Flag or Flag-NEURL1B. As shown in Fig. 1C, PDE9A co-localised with NEURL1 mainly in the cytoplasm in case of Flag-NEURL1, and in the cytoplasm and as well as at the plasma membrane in case of NEURL1-Flag, indicating that both the myristoylated and non-myristoylated forms of NEURL1 can efficiently localise to the cell membranes. In contrast, for Flag-NEURL1B only a minor overlap with PDE9A signal was detected in the plasma membrane whereas no discernible overlap was observed in the cytoplasm (Fig. 1C). Interestingly, while PDE9A, when overexpressed alone, appears to be mainly localised to the cell membranes (Fig. 1C), its localisation becomes markedly more cytoplasmic in the presence of NEURL1-Flag (Fig. 1C). This suggests that interaction of PDE9A with NEURL1 but not with NEURL1B could lead to translocation of the protein complex from the cell membrane to the cytosol. Altogether, our immunocytochemistry experiments indicate that PDE9A co-localises with NEURL1 to a greater extent than with NEURL1B.

We then asked whether the interaction between NEURL1 and PDE9A was specific or NEURL1 could also bind to other phosphodiesterases. To this end, we tested whether NEURL1 could interact with two other phosphodiesterases with broadly overlapping expression patterns in the nervous system, PDE5A ((hydrolysing both cAMP and cGMP^21^) and PDE11A ((hydrolysing only cGMP^21^). Co-immunoprecipitation experiments revealed that neither PDE11A nor PDE5A, unlike PDE9A, could interact with NEURL1 (Fig. 1D, lanes 5 and 6 *vs* lane 7). Thus, we conclude that the interaction between NEURL1 and PDE9A is specific.

### The interaction between NEURL1 and PDE9A is mediated by the NHR domains of NEURL1 and the catalytic and regulatory domains of PDE9A

**Figure 2:**
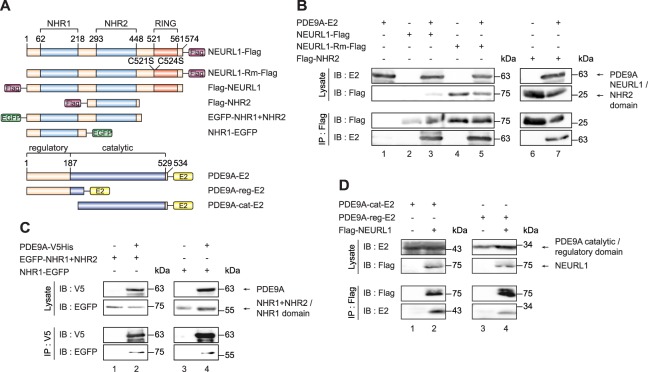
NEURL1-PDE9A interaction is mediated by the NHR1 and NHR2 domains of NEURL1 and the catalytic and regulatory domains of PDE9A. (**A**) Schematic diagram of the various NEURL1 and PDE9A constructs used. (**B**) NEURL1-Flag, NEURL1-Rm-Flag Flag (encoding the ubiquitin ligase defective protein), Flag-NHR2 and PDE9A-E2 were expressed in various combinations in HEK293-FT cells. (**C**) PDE9A-V5His protein was expressed alone or in combination with either EGFP-NHR1 + NHR2 or NHR1-EGFP in HEK293-FT cells. (**D**) PDE9A-cat-E2 or PDE9A-reg-E2 (encoding the catalytic and regulatory domains of PDE9A, respectively) were expressed alone or together with Flag-NEURL1 in HEK293-FT cells. In B-D the proteins were immunoprecipitated and immunoblotted using the indicated antibodies. NHR, Neuralized Homology Repeat; Rm, mutant RING domain; cat, catalytic domain; reg, regulatory domain; IB, immunoblot; IP, immunoprecipitation.

To identify the domains within NEURL1 mediating its interaction with PDE9A, we co-expressed various intact or mutant NEURL1 proteins (depicted schematically in Fig. 2A) and PDE9A in HEK293 cells and performed co-immunoprecipitation analyses. We found that NEURL1 proteins that have a mutated RING domain (Fig. 2B, lane 5) or lack it entirely but retain either both or one of the NHR domains (Fig. 2C, lanes 2, 4, 7) could immunoprecipitate PDE9A. We infer that both NHR domains independently, but not the RING domain mediate the interaction between NEURL1 and PDE9A.

To determine the PDE9A domains interacting with NEURL1, we expressed E2-tagged full-length PDE9A and its N-terminal and C-terminal halves (regulatory domain, amino acids M1-L228 and catalytic domain, amino acids M217-N534, respectively) with Flag-NEURL1 in HEK293 cells and performed immunoprecipitations using Flag antibody. Immunoblot analysis of the immunoprecipitates showed that both domains of PDE9A can independently bind NEURL1 (Fig. 2D, lanes 2 and 4).

### NEURL1 but not NEURL1B promotes polyubiquitination of PDE9A

**Figure 3:**
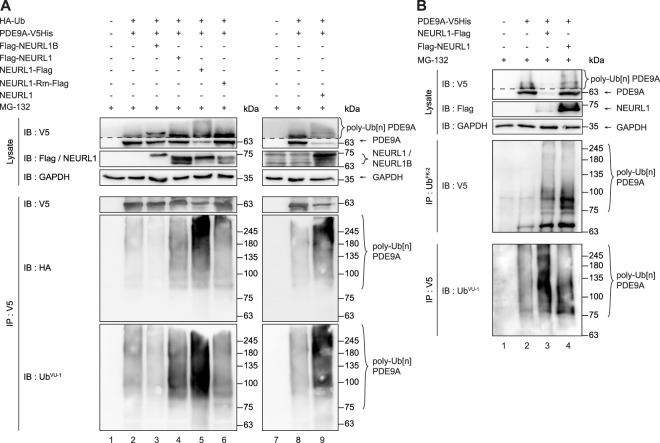
NEURL1 but not NEURL1B promotes ubiquitination of PDE9A. (**A**) HA-tagged Ubiquitin (HA-Ub), PDE9A-V5His, Flag-NEURL1B, Flag-NEURL1, NEURL1-Flag, NEURL1-Rm-Flag and untagged NEURL1 were expressed in various combinations in HEK293-FT cells in the presence of proteasomal inhibitor MG-132. The proteins were immunoprecipitated using the indicated antibodies. Exogenously and endogenously ubiquitinated PDE9A were detected with HA and Ub^VU-1^ antibodies, respectively. The higher molecular weight smear detected with HA and Ub^VU-1^ antibodies in lanes 4, 5 and 9 indicates polyubiquitination of PDE9A. (**B**) PDE9A-V5His was expressed alone or together with either NEURL1-Flag or Flag-NEURL1 in HEK293-FT cells. Following immunoprecipitation with the indicated antibodies, PDE9A was detected in immunoprecipitates of endogenous ubiquitin with V5 antibodies and endogenous ubiquitin was detected in PDE9A immunoprecipitate with Ub^VU-1^ antibodies. For clearer depiction of polyubiquinated PDE9A in the inputs of V5 immunoblots, the tonal range (i.e. levels) was changed differently for non-ubiquinated and polyubiquinated PDE9A species. The border for such differential level change is marked with a dotted line. IB, immunoblot; IP, immunoprecipitation.

NEURL1 has been shown to interact with and promote ubiquitination of several Notch ligands^7,10,11^, leading to their enhanced trafficking as well as degradation. NEURL1, however, also promotes ubiquitination of CPEB3 in a non-proteolytic fashion^13^. Thus, the observed interactions between NEURL1/NEURL1B and PDE9A suggested that they might also act as E3 ubiquitin ligases for PDE9A with either proteolytic or non-proteolytic consequences. To test this, we overexpressed both Flag-tagged and untagged NEURL1 or RING domain-mutant Flag-tagged proteins (Flag-NEURL1, NEURL1-Flag, untagged NEURL1 or NEURL1-Rm-Flag, respectively) and Flag-tagged NEURL1B with PDE9A-V5His and HA-tagged ubiquitin in HEK293-FT cells, then performed immunoprecipitation using V5 antibodies and subjected the immunoprecipitated proteins to immunoblotting using V5, HA and ubiquitin (Ub^VU-1^) antibodies. The latter two recognise exogenous or both exogenous and endogenous ubiquitin, respectively. We found that all intact NEURL1 proteins used (Flag-NEURL1, NEURL1-Flag and wild-type NEURL1) but not NEURL1B could promote ubiquitination of PDE9A with exogenous, HA-tagged ubiquitin, as evidenced by the presence of polyubiquitinated PDE9A species on immunoblots (Fig. 3A, lanes 4, 5, 9 *vs* lane 3). Consistent with previous reports^7,10^, we observed that an intact RING domain is required for the E3 ubiquitin ligase activity of NEURL1 since PDE9A was not ubiquitinated in the presence of RING-mutant NEURL1 (Fig. 3A, lane 6).

To determine whether NEURL1 is capable of promoting ubiquitination of PDE9A using endogenous ubiquitin, we overexpressed Flag-NEURLl, NEURL1-Flag and PDE9A-V5His alone or in various combinations in HEK293-FT cells and subjected the lysates to immunoprecipitation using either V5 or ubiquitin antibody Ub^FK-2^. Subsequent immunoblot analysis of the immunoprecipitated proteins using V5 or another ubiquitin antibody, Ub^VU-1^, revealed that PDE9A could be detected in ubiquitin immunoprecipitates and that PDE9A immunoprecipitates were polyubiquitinated. To test for the specificity of NEURL1-mediated polyubiquitination of PDE9A, we performed three control experiments similarly to those reported in Fig. 3A,B. We did not observe any readily discernible differences in polyubiquitination levels when either tagged (FLAG- NEURL1 and NEURL1-FLAG) or untagged NEURL1 or control plasmids pRC and pEGFP were overexpressed in HEK293-FT cells and detected using Ub^VU-1^ antibodies (Supplementary Fig. 3A). This implies that overexpression of NEURL1 does not lead to an overall, non-specific increase in the levels of endogenously polyubiquitinated proteins. In addition, we did not observe any obvious differences in the levels of exogenous polyubiquitination after overexpression of HA-tagged ubiquitin, both alone or in combination with tagged, untagged or RING-mutant (i.e ubiquitin ligase deficient) NEURL1 (Supplementary Fig. 3B). Finally, we also analyzed whether other V5-tagged proteins not known to interact with NEURL1 are ubiquitinated in the presence of NEURL1. Immunoblotting of the lysates of HEK293-FT cells overexpressing NEURL1-FLAG alone or with either V5-tagged PDE9A, TCF4, CREB or FOS as well as control plasmids pRC and pEGFP with V5 antibodies revealed a smear characteristic of polyubiquitiation in the case of PDE9A-V5 only (Supplementary Fig. 4).

Collectively, based on these results, we conclude that NEURL1 but not NEURL1B can promote ubiquitination of PDE9A.

### NEURL1 promotes polyubiquitination of PDE9A via ubiquitin’s lysine residues K27, K29 and K33

**Figure 4:**
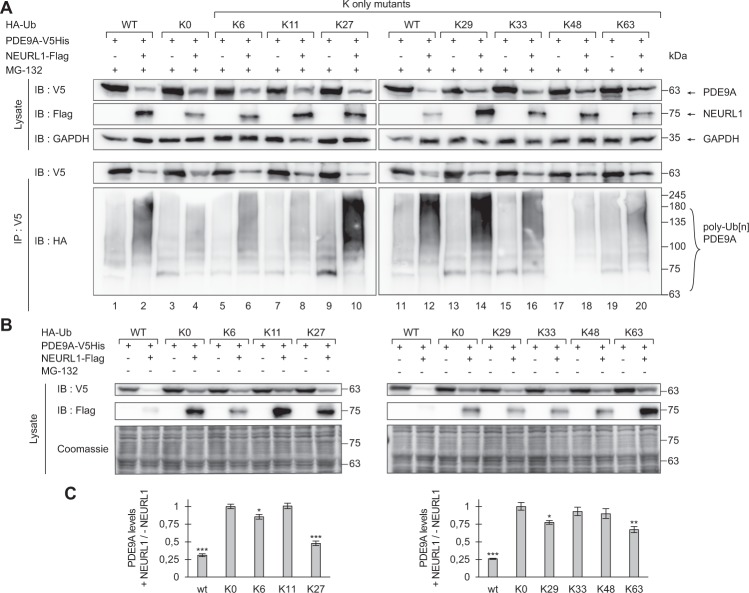
NEURL1 promotes polyubiquitination of PDE9A via lysines K27, K29 and K33 of ubiquitin. (**A**) Wild-type HA-tagged ubiquitin, K0 ubiquitin (i.e. ubiquitin mutant whose lysine residues had been substituted for arginine) and the various K only ubiquitin mutants were co-expressed with either PDE9A-V5His only or with PDE9A-V5His and NEURL1-Flag in HEK293-FT cells in the presence of proteasome inhibitor MG-132. The proteins were immunoprecipitated and immunoblotted using the indicated antibodies. (**B**) Wild-type HA-tagged ubiquitin, K0 ubiquitin and the various K only ubiquitin mutants were co-expressed with either PDE9A-V5His only or together with NEURL1-Flag in HEK293-FT cells in the absence of proteasome inhibitor MG-132. The lysates were immunoblotted using the indicated antibodies. (**C**) Quantification of NEURL1-mediated degradation of PDE9A in the presence of different ubiquitin mutants when overexpressed either alone or with NEURL1. Coomassie-normalised levels of PDE9A in cells co-expressing NEURL1 and the respective ubiquitin mutant were divided with the respective levels of PDE9A in cells where NEURL1 was not overexpressed. The calculated ratio in cells overexpressing the K0 mutant was arbitrarily set as 1. The average of 5 independent experiments is shown, with error bars representing SEM. Statistical significance was calculated relative to the ratio in cells expressing K0 ubiquitin. *p < 0.05, **p < 0.01, ***p < 0.001, two-tailed paired t-test. IB, immunoblot; IP, immunoprecipitation.

Since each of ubiquitin’s seven lysine residues (K6, K11, K27, K29, K33, K48, and K63) as well as its N-terminal methionine can be used to direct the formation of polyubiquitin chains on the substrate, formation of numerous different types of ubiquitin chains is possible. However, different K-linkages are usually associated with distinct cellular outcomes for a given substrate^20^. Thus, whereas ubiquitin chains conjugated to six out of the seven lysines (K6, K11, K27, K29, K33 and most commonly, K48) can target proteins for proteasomal degradation, K63 linked ubiquitin chains are associated with targeting proteins to endosomal-lysosomal system, DNA-damage responses and signalling processes leading to activation of NF-kappa-B^20^. Therefore, we aimed to identify the lysine residues of ubiquitin used for linkage of polyubiquitin chains on PDE9A. For this purpose, we co-expressed PDE9A, NEURL1 and exogenous wild-type ubiquitin or mutant ubiquitin where either all lysines or all but one had been mutated (K0 and K only mutants)^24^. In the K only mutants, only the single lysine available in the ubiquitin molecule can be used for its linkage to the growing polyubiquitin chain on the substrate. Following overexpression of PDE9A either alone or with NEURL1 in the presence of wild-type or K0 ubiquitin or the K-only mutants as well as the proteasome inhibitor MG-132, we immunoprecipitated PDE9A and subjected it to immunoblot analysis using HA antibodies. We found that PDE9A could not be polyubiquitinated when using the ubiquitin mutant where all of the lysines had been mutated or the K-only mutants where either K6, K11, K48 or K63 remained intact (Fig. 4, lanes 4, 6, 8, 18 and 20). In contrast, PDE9A was polyubiquitinated when using the K-only mutants where either K27, K29 or K33 were intact (Fig. 4A, lanes 10, 14 and 16), suggesting that these three lysine residues are utilised for NEURLl-mediated polyubiquitination of PDE9A.

We then overexpressed PDE9A either alone or with NEURL1 in the presence of wild-type or K0 ubiquitin or the K-only mutants in the absence of proteasome inhibitor MG-132 and subjected the lysates to immunoblot analysis using V5 or Flag antibodies (Fig. 4B). Quantification of PDE9A levels following its overexpression (Fig. 4C) showed significant reduction of PDE9A levels when co-expressed with NEURL1 in the presence of K27 and a minor reduction in the presence of either K6, K29 or K63 mutant as compared to PDE9A levels in the presence of K0 mutant. These results imply that the NEURL1-mediated degradation of PDE9A mainly requires the lysine residue K27 to form polyubiquitin chain linkages on PDE9A.

### NEURL1 mediates proteasomal degradation of PDE9A

**Figure 5:**
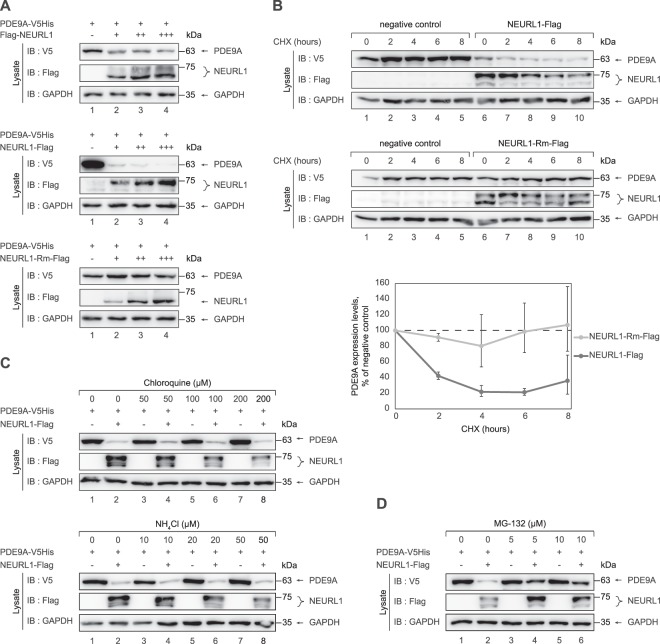
NEURL1 mediates proteasomal degradation of PDE9A in HEK293-FT cells. (**A**) PDE9A-V5His was expressed either alone or together with various amounts (−, 0 μg; + , 1 μg; ++, 2 μg; +++, 3 μg) of Flag-NEURL1 or NEURL1-Flag or NEURL1-Rm-Flag in HEK293-FT cells. 24 hours after transfection, the cells were lysed and the lysates were immunoblotted with the indicated antibodies. (**B**) PDE9A-V5His was co-expressed with empty vector (‘negative control’) or NEURL1-Flag or NEURL1-Rm-Flag in HEK293-FT cells. Following transfection, the cells were first incubated for 16 hours in the presence of proteasome inhibitor MG-132 and then treated with 100 µM of protein synthesis inhibitor cycloheximide (CHX) for the indicated time periods. The lysates were immunoblotted with the antibodies indicated and the protein levels from two independent experiments were quantified. (**C**,**D**) PDE9A-V5His was expressed alone or together with NEURL1-Flag in HEK293-FT cells. 24 hours after transfection, the cells were treated with lysosomal inhibitor chloroquine or ammonium chloride (**C**) or with proteasome inhibitor MG-132 (**D**) at the indicated concentrations for 24 hours. The cells were lysed and subjected to immunoblotting with the indicated antibodies. In all experiments shown in A through D, immunoblotting with anti-GAPDH antibody was performed to demonstrate equal loading. IB, immunoblot.

It has been reported that the levels of K27, K29 and K33 ubiquitin linkages – which we show are used during polyubiquitination of PDE9A – are low in the cells but they increase upon treatment of cells with proteasome inhibitors, implying that these linkages target certain ubiquitination substrates to the proteasome^25^. K29 linkages have been also associated with targeting the substrate proteins to the lysosomal degradation pathway^20^. This suggests that ubiquitination of PDE9A by NEURL1 could also result in reduction of PDE9A protein levels either via proteasomal or lysosomal degradation pathways. Alternatively, as shown for NEURL1-mediated ubiquitination of CPEB3^13^, ubiquitination of PDE9A by NEURL1 may not alter its protein levels and might therefore serve another, non-proteolytic function. To distinguish between these possibilities, we first tested whether co-expression of either Flag-NEURL1, NEURL1-Flag or NEURL1-Rm-Flag alters the levels of PDE9A-V5His in HEK293-FT cells. We found that PDE9A-V5His levels decreased when increasing amounts of Flag-NEURL1 and NEURL1-Flag-encoding vectors had been co-transfected (Fig. 5A, upper and middle panels, lanes 2–4). On the other hand, the levels of PDE9A remained unchanged when PDE9A was co-expressed with NEURL1-Rm-Flag that has no ubiquitin ligase activity (Fig. 5A, lower panel, lanes 2–4). This suggests that the presence of NEURL1 negatively influences the levels of PDE9A, either reducing its production or more likely enhancing its degradation. Hence, we next studied the time course of PDE9A accumulation and/or degradation quantitatively by analysing its levels in HEK293-FT cells when expressed either alone or with NEURL1-Flag or NEURL1-Rm-Flag in the presence of cycloheximide (CHX), a protein synthesis inhibitor. We found that when PDE9A was expressed alone, its levels did not change in response to treatment of the cells with cycloheximide (Fig. 5B, upper panel, lanes 2–5). In contrast, the levels of PDE9A became markedly reduced in the presence of NEURL1-Flag but not NEURL1-Rm-Flag (Fig. 5B, lanes 6–10, compare upper and lower panels). Such reduction of the PDE9A levels was apparent by two hours of cycloheximide treatment. These observations suggest that PDE9A is targeted for proteolysis following ubiquitination by NEURL1. The finding that the levels of NEURL1 also decrease in response to cycloheximide treatment (Fig. 5B, lanes 6–10 of the middle panel) is consistent with previous studies showing that NEURL1 can autoubiquitinate itself^10^, leading to a reduction of its protein levels.

Subsequently, we aimed to ascertain whether NEURL1-mediated degradation of PDE9A is achieved through proteasomal or lysosomal pathways. For this, we first overexpressed PDE9A either alone or together with NEURL1 and exposed the cells to increasing concentrations of either chloroquine or ammonium chloride, inhibitors of lysosomal function^26,27^. Neither chloroquine nor ammonium chloride treatment could reverse the NEURL1-mediated reduction of PDE9A levels (Fig. 5C, lanes 4, 6 and 8, upper and lower panels, respectively), showing that NEURL1 does not target PDE9A to the lysosomal pathway. We then performed a similar experiment using two different concentrations of MG-132, a proteasome inhibitor. We observed that the reduction of PDE9A levels by the presence of NEURL1 was partially reversed in cells that had been treated with MG-132 (Fig. 5D, lane 2 *vs* lanes 4 and 6).

**Figure 6:**
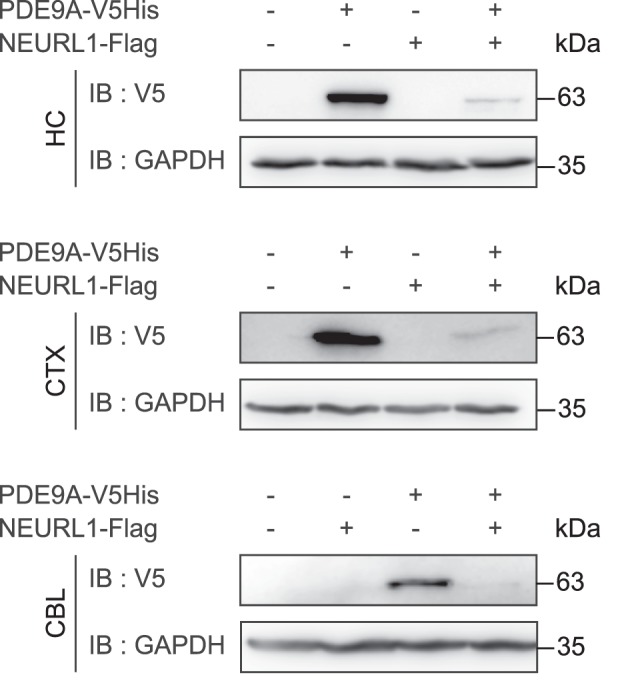
NEURL1 promotes degradation of PDE9A in primary neurons. PDE9A-V5His was expressed either alone or together with NEURL1-Flag in primary hippocampal (HC), cortical (CTX) or cerebellar (CBL) neurons. 2–3 days post transfection, the cells were lysed and subjected to immunoblotting with the indicated antibodies. NEURL1-Flag could not be unequivocally detected due to the emergence of high background signal in primary neurons when using anti-Flag antibodies (data not shown). Immunoblotting with anti-GAPDH antibody was performed to demonstrate equal loading. IB, immunoblot.

To ascertain whether the presence of NEURL1 also leads to a reduction in protein levels of PDE9A in neuronal cells, we overexpressed PDE9A-V5His either alone or together with NEURL1-FLAG in rat primary hippocampal, cortical and cerebellar neurons and determined the levels of both proteins thereafter. Since, like in HEK293-FT cells, the levels of PDE9A became markedly reduced when co-expressed with NEURL1-Flag in all three primary neuronal cell types (Fig. 6, lanes 2 *vs* 4 in upper and middle panels and lanes 3 *vs* 4 in the lower panel), we conclude that NEURL1-mediated down-regulation of PDE9A also occurs in primary neurons. The latter suggests that NEURL1-mediated targeting of PDE9A for degradation is a universal phenomenon and not just confined to one cell type. Collectively, the above experiments indicate that NEURL1-mediated ubiquitination results in proteasomal degradation of PDE9A.

## Discussion

Several studies have shown that Neuralized interacts with and promotes ubiquitination of Notch ligands and CPEB3^7,10,11,13^ with both proteolytic and non-proteolytic outcomes. In our yeast two-hybrid screen for novel NEURL1 interaction partners, one of the proteins uncovered was a cGMP-specific PDE9A. Cyclic nucleotide phosphodiesterases are enzymes hydrolysing the phosphodiester bond in cAMP and/or cGMP, intracellular secondary messengers implicated in various physiological and pathological processes^21^. Human *PDE9A* has multiple isoforms and is expressed in the brain, small intestine, heart and spleen^28^. Rat *PDE9A* is highly expressed in the brain, particularly in forebrain, cerebellum and olfactory bulb^23^. *NEURL1* and *NEURL1B* are also highly expressed in the same regions of adult brain^11,14^.

We thus sought to confirm whether PDE9A might constitute a novel interactor and a substrate for NEURL1 and NEURL1B by co-immunoprecipitation, subcellular co-localisation and ubiquitination experiments using overexpressed proteins. Both NEURL1 and NEURL1B interacted with PDE9A in co-immunoprecipitation experiments, however, NEURL1 immunoprecipitated significantly more PDE9A than NEURL1B. Our finding that both NHR domains but not the RING domain of NEURL1 interact with PDE9A is consistent with previous studies demonstrating that the RING domain has the E3 ubiquitin ligase activity while the NHR domains interact with substrates^10,29,30^. The observed NEURL1-PDE9A interaction appears specific, since we did not detect any interaction between overexpressed NEURL1 and two other phosphodiesterases, PDE5A (cGMP-specific) or PDE11A (cAMP/cGMP dual-specific).

We attempted to test whether NEURL1 and PDE9A also interact endogenously. However, since none of the PDE9A antibodies that we tested could immunoprecipitate PDE9A or recognise it on immunoblots, this remains currently unsolved. Nonetheless, using a custom-raised polyclonal antibody against NEURL1, we were able to demonstrate that both endogenous and overexpressed untagged NEURL1 could interact with tagged PDE9A in a co-immunoprecipitation experiment in HEK293-FT cells. This finding lends support to the possibility of an endogenous interaction between NEURL1 and PDE9A.

NEURL1/NEURL1B and PDE9A interactions imply that the subcellular distribution of these proteins should overlap at least partially. While, to our knowledge, the subcellular localisation of rat PDE9A has not been reported, human PDE9A splice variant hPDE9A2, corresponding to the rat PDE9A used in this study, is localised both in the cell membrane and cytoplasm^28^. Our immunofluorescence analysis showed a considerable overlap in the localisation of NEURL1 and PDE9A and a minor co-localisation of NEURL1B and PDE9A, consistent with their interaction in co-immunoprecipitation experiments. Overexpressed NEURL1 and PDE9A were both localised predominantly in the cytoplasm and, to a lesser extent, in the cell membranes. On the other hand, localisation of NEURL1B and PDE9A overlapped mostly in the cytoplasm, and to a lesser degree, in the cell membranes. We also observed that, in the presence of NEURL1, subcellular localisation of PDE9A becomes predominantly cytoplasmic compared to its mainly cell membrane-associated distribution when overexpressed alone. This implies that upon interacting with NEURL1, PDE9A becomes partially localised from the cell membranes to the cytosol. Alternatively, it is also possible that the interaction with NEURL1 retains PDE9A in the cytoplasm and prevents PDE9A from localising to the plasma membrane.

Interestingly, we found that both the catalytic and regulatory domains of PDE9A could interact with NEURL1. While there is no data on the stoichiometry of the NEURL1-PDE9A interaction, these findings suggest that one molecule of NEURL1 can bind two molecules of PDE9A and vice versa. Alternatively, it is also feasible that while one NHR domain interacts with the catalytic domain of PDE9A, the other one interacts with PDE9A’s regulatory domain.

Since NEURL1 promotes ubiquitination of Notch ligands and CPEB3^7,10,11,13^, we sought to analyse whether PDE9A could also constitute its substrate. The finding that only wild-type but not RING-mutant NEURL1 can promote ubiquitination of PDE9A shows that NEURL1 can also function as an E3 ligase for PDE9A. In contrast, we found that NEURL1B could not promote ubiquitination of PDE9A in spite of being its interacting partner. This finding, coupled with the observation that PDE9A partially relocalises from the cell membranes to the cytoplasm in the presence of NEURL1 but not NEURL1B implies that relocaliation of PDE9A probably depends upon its polyubiquitination by NEURL1.

Polyubiquitination can be achieved via using any of the ubiquitin’s seven lysine residues to assemble the ubiquitin moieties into a polyubiquitin chain. The ubiquitin linkages utilised on different substrates are distinct, and their nature determines whether ubiquitination of a given substrate serves a proteolytic or non-proteolytic function^20^. To date, the K63 linkage is the only one that has been shown not to be involved in targeting of ubiquitinated proteins for proteasomal degradation^20,25^. Our finding that three different K linkages used for NEURL1-mediated ubiquitination of PDE9A are K27, K29 and K33 and that NEURL1-mediated degradation of PDE9A mainly requires the lysine residue K27 of ubiquitin suggest that PDE9A is targeted for proteolysis.

Our subsequent experiments showed that NEURL1-mediated ubiquitination of PDE9A does indeed serve a proteolytic function, via a proteasome-based mechanism. Firstly, the protein levels of PDE9A were markedly reduced when co-expressed with wild-type NEURL1 in both HEK293-FT and primary cerebellar, cortical and hippocampal neurons. In contrast, the levels of PDE9A remained unchanged when co-expressed with the RING-mutant NEURL1 in HEK293-FT cells indicating that only ubiquitinated PDE9A can be targeted for proteolysis. Secondly, the protein accumulation assay revealed that the protein levels of PDE9A remained unchanged following cycloheximide treatment when PDE9A had been expressed alone or with RING mutant PDE9A, but not with wild-type NEURL1. Since the PDE9A levels decreased in a time-dependent manner in the presence of NEURL1, degradation but not *de novo* production of PDE9A must be affected by NEURL1. Thirdly, experiments with inhibitors of either lysosomal or proteasomal function unequivocally demonstrated that NEURL1 targets PDE9A for proteasomal but not lysosomal destruction. This finding is consistent with the fact that, frequently, soluble proteins are degraded by the proteasome whereas membrane-bound proteins, including other known substrates of NEURL1 such as Notch ligands are recycled by the endosome-lysosome pathway^10^.

The physiological role of the interaction of NEURL1 and PDE9A is not known. However, it has been shown that in mice NEURL1 is required for synaptic plasticity and memory storage^13^. Furthermore, it has also been shown that cGMP is required for both synaptic depression and potentiation in the hippocampus^31,32^. Since both NEURL1^14^ and PDE9A^23^ are expressed in hippocampus, it is plausible that the interaction between NEURL1 and PDE9A is important for synaptic plasticity by upregulating cGMP levels via NEURL1-mediated proteolytic ubiquitination of PDE9A. Future studies are required to confirm this scenario.

Only a handful of ubiquitin ligases targeting phosphodiesterases have been described so far^33,34^. Our study is the first to report an E3 ubiquitin ligase for PDE9A. Our results suggest that NEURL1 could regulate PDE9A by targeting it for proteasome-mediated degradation mainly via lysine residue K27 of ubiquitin and thereby possibly positively affect cellular cGMP levels.

## Methods

### Expression constructs

Plasmids pFlag-NEURL1, pFlag-NEURL1B, pEGFP-NEURL1, rNEURL1-pcDNA3 have been described previously^10,11,14,35^. The following plasmids were purchased from Addgene^24^: pNEURL1-Flag (plasmid #17282), pNEURL1-Rm-Flag (plasmid #17319), pFlag-NHR2 (plasmid #17318), pRK5-HA-Ubiquitin-WT (plasmid #17608), pRK5-HA-Ubiquitin-K0 (plasmid #17603), pRK5-HA-Ubiquitin-K6 (plasmid #22900), pRK5-HA-Ubiquitin-K11 (plasmid #22901), pRK5-HA-Ubiquitin-K27 (plasmid #22902), pRK5-HA-Ubiquitin-K29 (plasmid #22903), pRK5-HA-Ubiquitin-K33 (plasmid #17607), pRK5-HA-Ubiquitin-K48 (plasmid #17605), pRK5-HA-Ubiquitin-K63 (plasmid #17606). pEGFP-NHR1 + NHR2 was generated by removing sequences downstream of NHR2 domain from the parental plasmid pEGFP-NEURL1 using *Bst*XI restriction enzyme (Thermo Fisher Scientific). All other expression constructs used were generated by RT-PCR-based cloning using the cDNAs and oligonucleotides described in Supplementary Table 1. Full-length rat PDE9A coding region was cloned into pcDNA3.1/V5-His-TOPO (Invitrogen Life Technologies) and pQM-E2tag vectors (Icosagen). pPDE9A-reg-E2 and pPDE9A-cat-E2, coding for the N-terminally located regulatory domain (amino acids M1-L228) and the C-terminal catalytic domain (amino acids M217-N534) of PDE9A, respectively, were PCR-cloned from the resulting pPDE9A-E2 plasmid into pQM-E2tag. Mouse PDE5A and PDE11A full-length coding region cDNAs were cloned into pQM-E2tag.

### Yeast two hybrid screening

Yeast two-hybrid screening was performed as described in^36^. pYTH6-mNeurl1 was linearised and integrated into the yeast genome of yeast strain Y190 by a lithium acetate/polyethylene glycol-based transformation method and used to screen a rat adult brain cDNA library (Clontech).

### Cell culture, transfections and immunofluorescence analysis

For immunofluorescence analysis, HEK293 cells (purchased from ATCC; catalog number: ATCC® CRL-1573™) were grown, transfected and prepared for imaging analysis as described in^11,37^. Immunofluorescence analysis was performed with Zeiss LSM Duo confocal microscope. For immunoprecipitation and ubiquitination assays, HEK293-FT cells (purchased from Thermo Fisher Scientific; catalog number: R70007) were grown and transfected as described in^11,37^.

### Antibodies, immunoprecipitation and immunoblotting

The sources and concentrations of antibodies and antibody-conjugated agarose beads used are listed in Supplementary Table 2. Affinity-purified rabbit polyclonal antibodies raised against the peptide CRRPIKDIIKTYRSS (synthesised by InBio), corresponding to the C-terminal region of mNEURL1 (amino acids 560–574), were generated by LabAS.

Immunoprecipitation and immunoblotting was essentially performed as described by^11,37^. Briefly, HEK293**-**FT cells expressing untagged or Flag-tagged NEURL1 and V5His- or E2-tagged PDE9A were lysed in lysis buffer containing 20 mM Tris HCl (pH 7.4), 200 mM NaCl, 1 mM EDTA, 0.5% NP-40 and protease inhibitor cocktail (Roche) for 30 min, sonicated and then centrifuged for 10 min at maximum speed at +4 °C. 1–2 mg of total protein lysate was used for each immunoprecipitation. To immunoprecipitate V5His-tagged PDE9A or Flag-tagged NEURL1 or NEURL1B, the lysates were incubated overnight at +4 °C with 40 μL of agarose slurry (Sigma) that had been conjugated with V5 or Flag antibodies. To immunoprecipitate untagged NEURL1 or E2-tagged PDE9A, 40 μL of Protein A sepharose bead slurry (GE Healthcare) that had been conjugated with either E2 or NEURL1 antibodies were used. The V5-agarose, Flag-agarose or Protein A sepharose beads bound to the immunoprecipitated proteins were washed 5x for 10 min with lysis buffer at +4 °C. Immunoprecipitated proteins were eluted using 2x Sample Buffer and 20% beta-mercaptoethanol and subjected to immunoblot analysis as in^11,37^.

### Immunoprecipitation of ubiquitinated proteins

The constructs encoding Flag-NEURL1, Flag-NEURL1B, NEURL1-Flag, NEURL1-Rm-Flag, PDE9A-V5His and plasmids carrying HA-tagged wild-type ubiquitin or HA-tagged ubiquitin with no lysines or K-only ubiquitin mutants were overexpressed in various combinations in HEK293-FT cells. Wild type ubiquitin can use any of its seven lysines for ubiquitin polymerisation. The other six lysines of each of the K-only mutant ubiquitin constructs had been mutated to arginine, and therefore could not serve as substrates for ubiquitin polymerisation. Instead, they can only polymerise using one of the seven lysines that is not mutated into arginine^24^. 24 hours after transfection, 5 μM of proteasome inhibitor MG-132 (Sigma) was added to the transfected cells and 24 hours later the cells were lysed in a lysis buffer containing 50 mM Tris-HCl (pH 8.0), 150 mM NaCl, 1 mM EDTA, 1% Triton X-100, 1% Na-deoxycholate, 0.1% SDS and protease inhibitor cocktail (Roche). Lysates containing 1–2 mg of total protein were then incubated overnight at +4 °C by rotating with 40 μL of agarose slurry that had been conjugated with V5 antibodies (Sigma). Alternatively, lysates were first incubated with ubiquitin Ub^FK-2^ antibodies (ENZO Life Sciences) for 2 hours at +4 °C by rotation. 40 μl of Protein G Dynabeads (Thermo Fisher Scientific) were then added to the lysate-antibody mixture and incubated for 2 hours at +4 °C by rotation. The beads with bound proteins were washed 5x for 10 min at +4 °C in lysis buffer and the proteins were eluted using 2x Sample Buffer and 20% beta-mercaptoethanol and analysed with immunoblotting using V5 antibodies or ubiquitin antibody Ub^VU-1^ (LifeSensors) as in^11,37^.

### Protein accumulation and degradation assays

To analyse the effects of different concentrations of NEURL1 on the protein levels of PDE9A HEK293-FT cells were transfected with 1, 2 of 3 µg of Flag-NEURL1 or NEURL1-Flag and 1 µg of PDE9A constructs. Following of 48 hours of co-expression, the cells were lysed in a lysis buffer containing 4 M urea, 20 mM Tris-HCl (pH 7.5), 135 mM NaCl, 1% Triton X-100, 10% glycerol and 1.5 mM MgCl_2_ and subjected to immunoblot analysis as described above. Equal amounts of total protein (20–30 µg) were loaded onto each lane. Equal loading of total protein was verified by immunoblot-based analysis of GADPH levels using the respective antibody.

For protein accumulation and degradation experiments, HEK293-FT cells overexpressing either NEURL1 or PDE9A alone or simultaneously were treated with the lysosomal inhibitors chloroquine (Sigma) and NH_4_Cl (Scharlau) or the proteasomal inhibitor MG-132 (Sigma), or the translational inhibitor cycloheximide (Tocris). Both lysosomal and proteasomal inhibitors were added to the cells 24 hours after transfection for the duration of 24 hours.

Half-life of the NEURL1 and PDE9A proteins were measured in HEK293-FT cells overexpressing either NEURL1 and PDE9A or NEURL1-Rm-Flag and PDE9A essentially as described by^38^. Briefly, following transfection, the cells were grown in the presence of 5 µM MG-132 for 16 hours, the media was then replaced with the one containing 100 µM of cycloheximide for 0, 2, 4, 6 or 8 hours. The lysates were separated on SDS-PAGE, immunoblotted with the antibodies indicated and the protein levels were subsequently quantified using ImageQuant Las 4000 and and ImageQuant TL software (GE Healthcare). For quantification of immunoblot data, the expression levels of all proteins were first normalised to those of GAPDH levels of the same sample as determined by using the respective antibody (Millipore). The normalised expression levels of PDE9A at different time points were then normalised against the corresponding time points of PDE9A expression levels when expressed alone (i.e. in the absence of either NEURL1-Flag or NEURL1-Rm-Flag), and plotted using Microsoft Excel software.

### Primary neuronal cultures

The protocols involving animals were approved by the ethics committee of animal experiments at Ministry of Agriculture of Estonia (Permit Number: 45). All experiments were performed in accordance with the relevant guidelines and regulations. Sprague-Dawley female and male rats were mated and pregnant females were euthanised using CO_2_ gas chamber after 20 to 21 days of gestation (E20-21). Primary cortical and hippocampal neuron cultures were prepared from male and female E20–21 rat pups essentially as described in^39^. Primary cerebellar neurons were derived using a similar protocol from P7 rat pups, and cultivated in medium that was supplemented with additional KCl, bringing its final concentration to 25 mM.

Primary cortical and hippocampal neurons were transfected on the day of culture derivation using the Rat Neuron Nucleofector® Kit (Lonza) according to the manufacturer’s instructions. The transfected primary cortical and hippocampal neurons were lysed on the third day *in vitro* (DIV) directly into 2x Laemmli Buffer that had been supplemented with 10% beta-mercaptoethanol. Primary cerebellar neurons were transfected using Lipofectamine® 2000 (Thermo Fisher Scientific) for 4 hours at 6 DIV according to the manufacturer’s instructions and were lysed at 8 DIV. In all co-transfections, the ratio of NEURL1 and PDE9A plasmids was 1:1.

## Supplementary information

Supplementary information
