# Supplementary material for: Neuralized family member NEURL1 is a ubiquitin ligase for the cGMP-specific phosphodiesterase 9A
Source: Sci Rep. 2019 May 8;9:7104. doi: 10.1038/s41598-019-43069-x (PMC6506465; doi:10.1038/s41598-019-43069-x)
Supplement: Supplementary file 1 — Supplementary information [file 41598_2019_43069_MOESM1_ESM.pdf]

**Neuralized family member NEURL1 is a ubiquitin ligase for the cGMP-specific phosphodiesterase 9A**

Kati Taal, Jürgen Tuvikene, Grete Rullinkov, Marko Piirsoo, Mari Sepp, Toomas Neuman, Richard Tamme, Tõnis Timmusk

**Table 1.** Primers for RT-PCR-based cloning of expression constructs used in this study.

| Species | Cloned gene | cDNA source | Oligonucleotide sequence |
|---|---|---|---|
| rat | PDE9A | hippocampus (adult) | (fw) CCACCATGGGGGCCGGCTCCTCAA<br>(rev) GTTTGGAGGCGAACGGTCTT |
| rat | PDE9A regulatory domain | full-length PDE9A | (fw) CCACCATGGGGGCCGGCTCCTCAA<br>(rev) CAGACCAAGGTCATGGTACAT |
| rat | PDE9A catalytic domain | full-length PDE9A | (fw) CACCATGTACCATGACCTTGGTCTG<br>(rev) GTTTGGAGGCGAACGGTCTT |
| mouse | PDE5A | testis (adult) | (fw) CTGTCTAGAATGGAACGAGCGGGCCCC<br>(rev) CTGCCTGCAGGGTCCCGCTTGCCCTGGCT |
| mouse | PDE11A | hippocampus (adult) | (fw) CTGGGATCCATGGCAGCCTCCCGCCTG<br>(rev) CTGAAAGCTTCAGTCTGTCTTCCCCGGCCACC |

**Table 2.** Antibodies and antibody-based reagents used in this study.

| Antibody | Dilution/concentration | Source/comments/public identifier[1] |
|---|---|---|
| **Primary antibodies:** | | |
| mouse monoclonal antiFlag M2-HRP | 1:5000 (IB) | Sigma-Aldrich, Cat# A8592, RRID:AB_439702 |
| rabbit polyclonal antiFlag | 5 µg/ml (ICC) | Sigma-Aldrich, Cat# F7425, RRID:AB_439687 |
| mouse monoclonal antiV5 | 1:5000 (IB),1:200 (ICC) | Invitrogen Life Technologies, Cat# R960CUS, RRID:AB_159298 |

| | | |
|---|---|---|
| mouse monoclonal antibody raised against Bovine Papillomavirus type 1E2-protein | 1:5000 (IB),1:1000 (IP) | Icosagen AS, Cat# A-100-100, RRID:AB_11133493 |
| rabbit polyclonal anti-EGFP | 1:200000 (IB) | described in Tamberg *et al.*[2] |
| mouse monoclonal anti-GAPDH | 1:5000 (IB) | Millipore, Cat# MAB374, RRID:AB_2107445 |
| mouse monoclonal antiubiquitin FK2 | 1:1000 (IB) | Enzo Life Sciences, Cat# BMLPW8810, RRID:AB_10541840 |
| mouse monoclonal antiubiquitin VU-1 | 1:1000 (IB) | LifeSensors, Cat#VU101 |
| rabbit polyclonal anti-NEURL1 | 1:100 (IB), 1:100 (IP) | generated by LabAS using the peptide CRRPIKDIIKTYRSS (corresponding to aa 560-574 of NEURL1) synthesised by Inbio |
| **Secondary antibodies:** | | |
| Alexa Fluor 546conjugated goat antirabbit IgG (H+L) | 1:2000 | Invitrogen Life Technologies, Cat# A11071, RRID:AB_1500774 |
| Alexa Fluor 488conjugated goat antimouse IgG (H+L) | 1:2000 | Invitrogen Life Technologies, Cat# A11017, RRID:AB_143160 |
| goat HRP-conjugated anti-mouse IgG (H+L) | 1:5000 | Thermo Scientific, Cat# 32430, RRID:AB_1185566 |
| | | |
| **Antibody-conjugated agarose beads:** | | |
| anti-Flag M2 Affinity Gel | 40 µl of gel suspension per reaction | Sigma-Aldrich, Cat# A2220, RRID:AB_10063035 |
| anti-V5 Agarose Affinity Gel | 40 µl of gel suspension per reaction | Sigma-Aldrich, Cat# A7345, RRID:AB_10062721 |
| | | |

[1] Public identifier from the Antibody Registry (www.antibodyregistry.org)

[2] N. Tamberg, V. Lulla, R. Fragkoudis, A. Lulla, J.K. Fazakerley, A. Merits, Insertion of EGFP into the replicase gene of Semliki Forest virus results in a novel, genetically stable marker virus. *J Gen Virol* 88 (Pt 4) (2007) 1225-30.

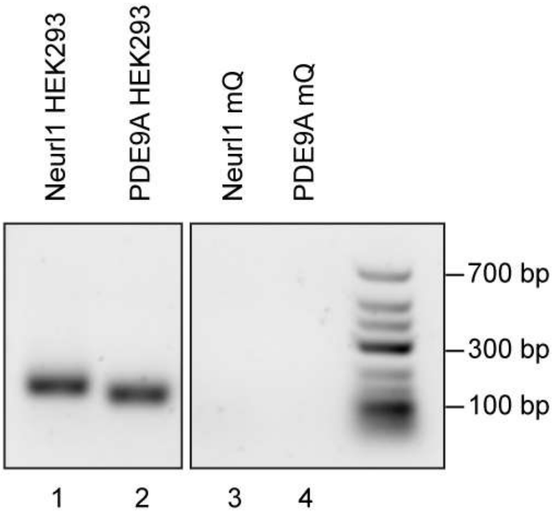


**SUPPLEMENTARY FIGURE 1. Analysis of *NEURL1* and *PDE9A* mRNA expression in HEK293 cells.** PCR analysis of *NEURL1* and *PDE9A* expression using cDNAs derived from HEK293 cells. *NEURL1* and *PDE9A* mRNAs are present in this cell line (lanes 1 and 2).

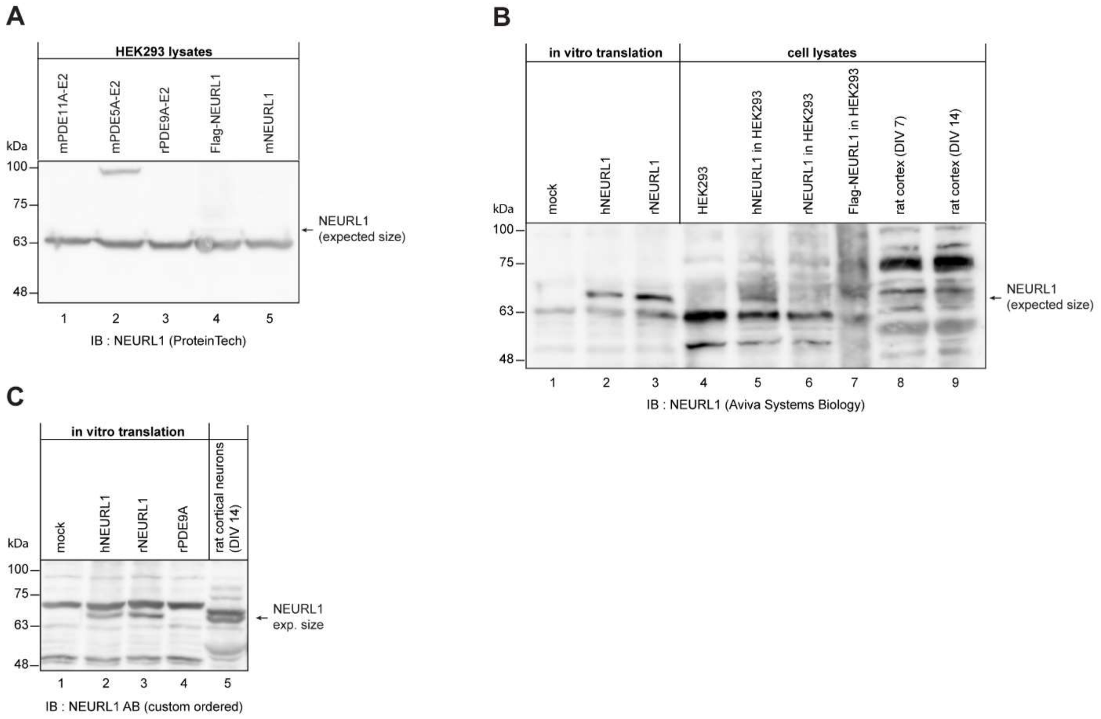


**SUPPLEMENTARY FIGURE 2. Testing the specificity and sensitivity of polyclonal ProteinTech, AVIVA Systems Biology and custom-ordered NEURL1 antibodies for detecting exogenous and endogenous NEURL1.**

(A) E2-tagged mouse PDE911A (mPDE911A), mouse PDE5A (mPDE5A), rat PDE9A (rPDE9A), Flag-tagged and untagged mouse NEURL1 (mNEURL1) were expressed in HEK293 cells and immunoblotted with NEURL1 antibody from ProteinTech (catalogue #PD9A-101AP) according to manufacturer's instructions. In vitro translated untagged human NEURL1 (hNEURL1) and rat NEURL1 (rNEURL1, lanes 2 and 3) were used as positive controls to visualise the expected size of NEURL1. HEK293 cells expressing E2-tagged mPDE911A, mPDE5A, rPDE9A were used as negative controls to detect the possible nonspecific binding of the NEURL1 antibody. ProteinTech's NEURL1 antibody only recognises a protein whose molecular size is smaller than expected for NEURL1 in both lysates from HEK293 cells expressing various PDE proteins (lanes 1-3) and tagged or untagged NEURL1 (lanes 4 and 5). It also appears to non-specifically recognise a protein with a molecular weight corresponding to that of PDE5A (lane 2). Thus, this antibody does not recognise either exogenous or endogenous NEURL1.

(B) In vitro translated hNEURL1 and rNEURL1, lysates of HEK293 cells overexpressing various tagged NEURL1 proteins and rat primary cortical neurons were immunoblotted with NEURL1 from Aviva Systems Biology (#ARP45760_P050) according to manufacturer's instructions. This antibody recognises in vitro translated NEURL1 proteins (lanes 2 and 3), but not overexpressed NEURL1 protein (lanes 5, 6 and 7). It also does not recognise endogenous NEURL1 either from the lysates of HEK293 cells or primary cortical neurons (lanes 4, 8 and 9).

(C). In vitro translated hNEURL1, mNEURL1, rPDE9A and lysates from rat primary cortical neurons proteins were immunoblotted with custom-ordered NEURL1 from LabAS. In vitro translated rPDE9A was used as a negative control (lane 4). This antibody recognises both in vitro translated hNEURL1 and mNEURL1 proteins (lanes 2 and 3) and endogenous NEURL1 from rat cortical neurons (lane 5). Abbreviations, IB – immunoblot; DIV – days in vitro.

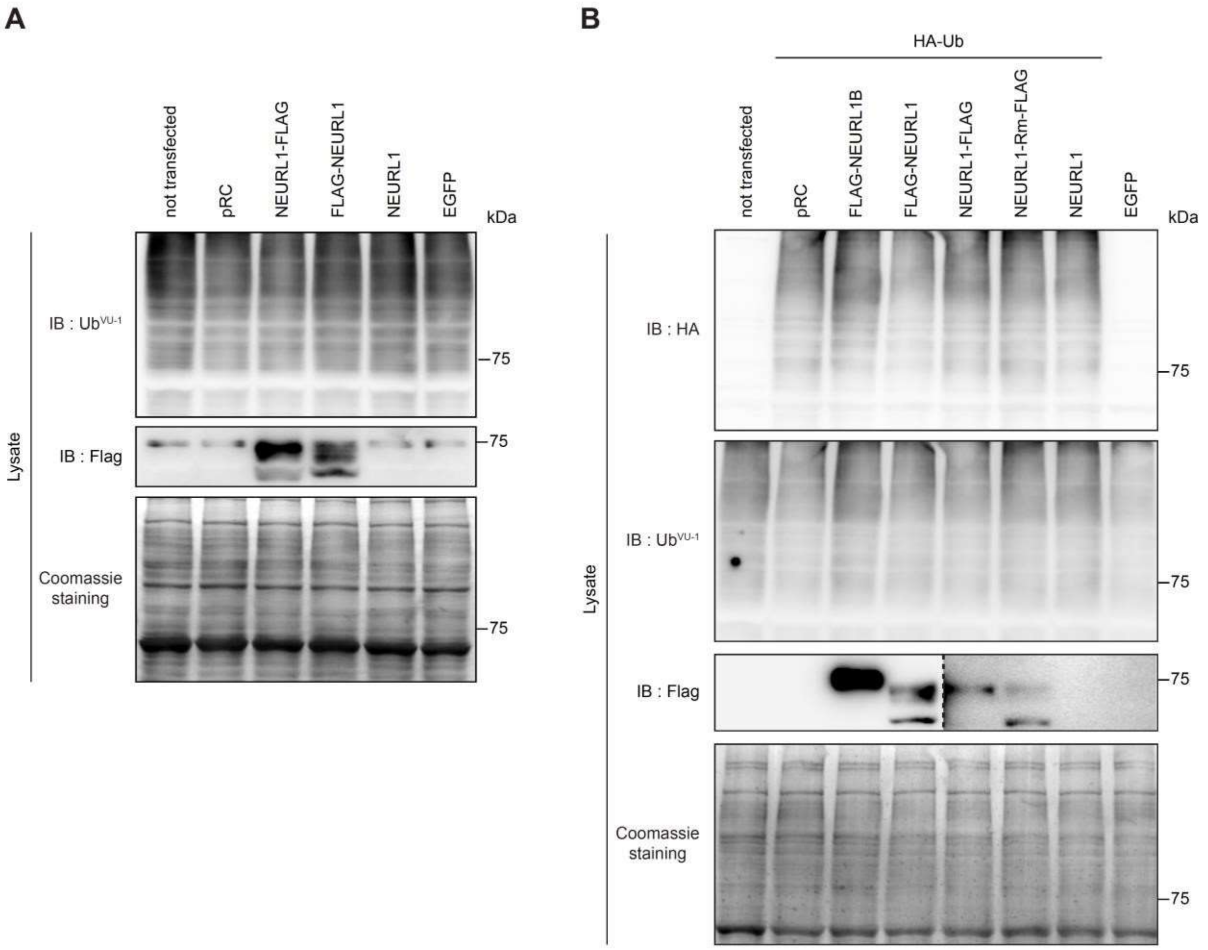


**SUPPLEMENTARY FIGURE 3. NEURL-mediated ubiquitination of PDE9A-V5 does not lead to an overall increase of endogenous or exogenous ubiquitination.** The indicated proteins were over-expressed in various combinations in the absence (A) or presence (B) of exogenous, HA-tagged ubiquitin in HEK293-FT cells and the lysates were subjected to immunoblotting with FLAG or HA or $Ub^{VU\text{-}1}$ antibodies. The membranes were stained with Commassie Brillant Blue to ensure equal loading.

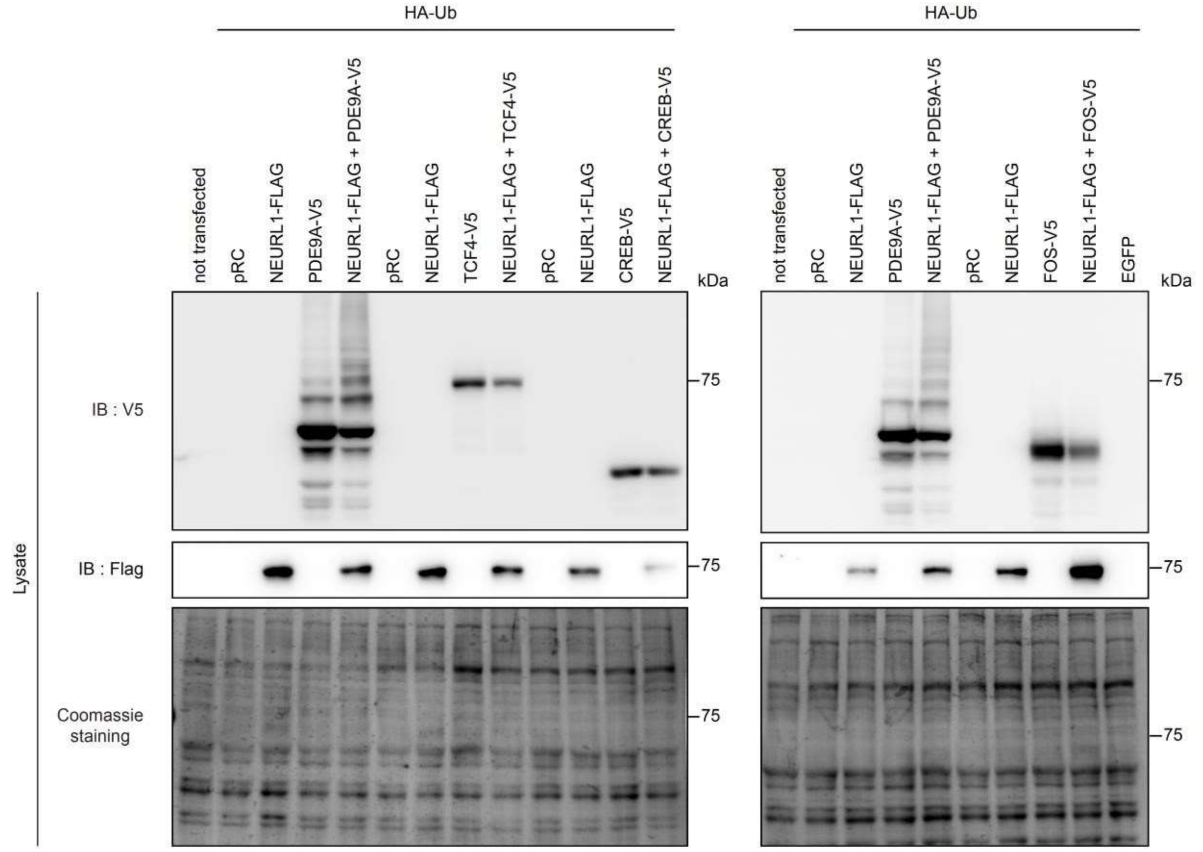


**SUPPLEMENTARY FIGURE 4. NEURL1 specifically promotes ubiquitination of PDE9A-V5, but not other V5-tagged proteins.** The indicated proteins were over-expressed in various combinations in the presence of exogenous, HA-tagged ubiquitin in HEK293-FT cells and the lysates were subjected to immunoblotting with V5 or FLAG antibodies. A smear indicating polyubiquitination is evident in the case of PDE9A-V5 but not CREB-V5, FOS-V5 or TCF4-V5. Since a weaker smear corresponding to polyubiquitination was also observed when PDE9A-V5 was over-expressed alone, we infer that while endogenous NEURL1 and other ubiquitin ligases promote polyubiquitination of PDE9A, exogenous NEURL1 greatly enhances the efficiency of this process. The membranes were stained with Commassie Brillant Blue to ensure equal loading.

## Uncropped Western blot images displayed in the main figures

# Figure 1A

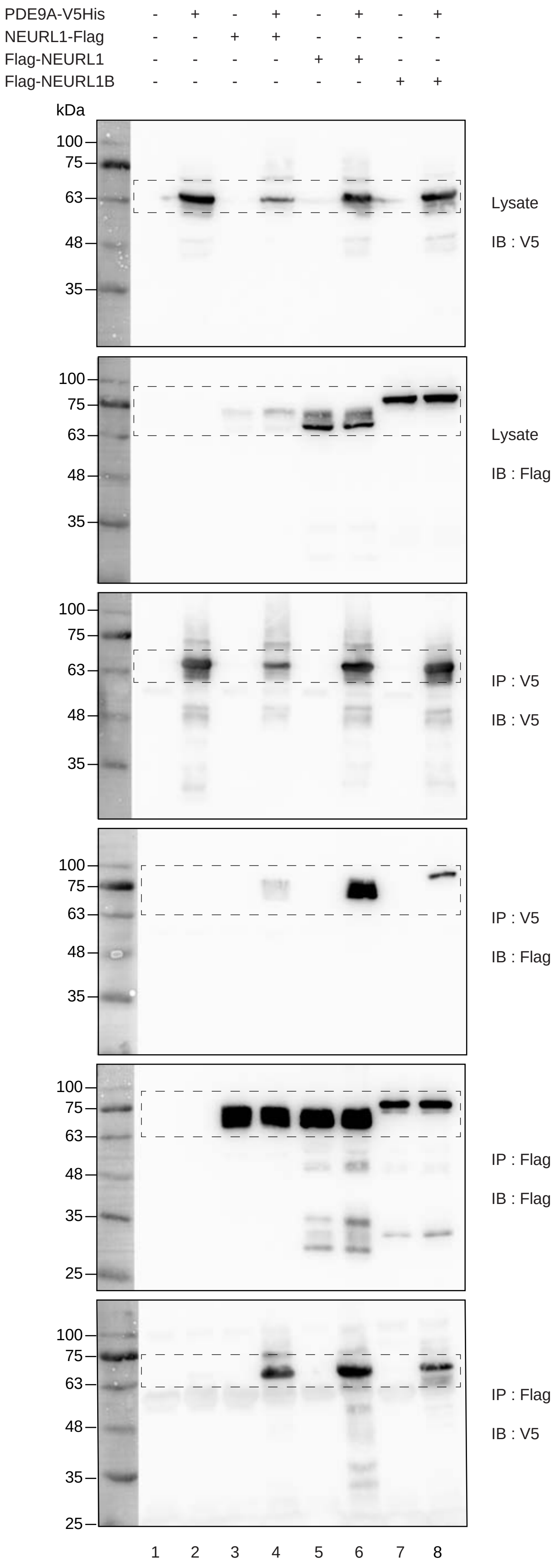

PDE9A-V5His - + - + - + - +
NEURL1-Flag - - + + - - - -
Flag-NEURL1 - - - - + + - -
Flag-NEURL1B - - - - - - + +
kDa
100
75
63
48
35
25
Lysate
IB : V5
Lysate
IB : Flag
IP : V5
IB : V5
IP : V5
IB : Flag
IP : Flag
IB : Flag
IP : Flag
IB : V5
1 2 3 4 5 6 7 8

# Figure 1B

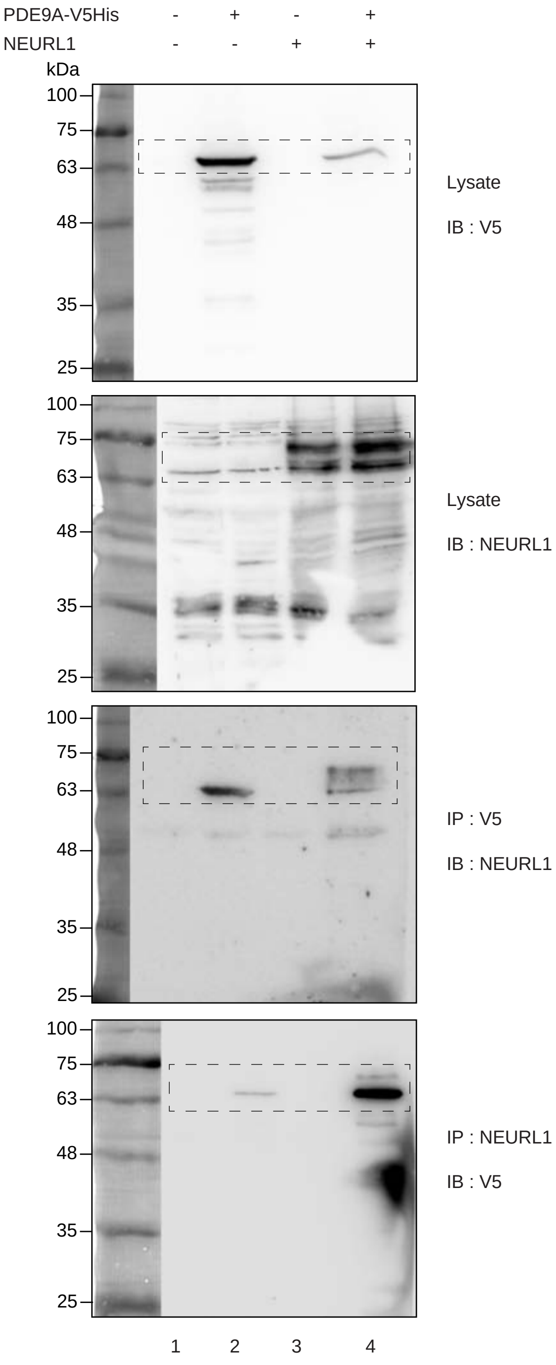
PDE9A-V5His - + - +
NEURL1 - - + +
kDa
100
75
63
48
35
25
Lysate
IB : V5
Lysate
IB : NEURL1
IP : V5
IB : NEURL1
IP : NEURL1
IB : V5
1 2 3 4

# Figure 1D

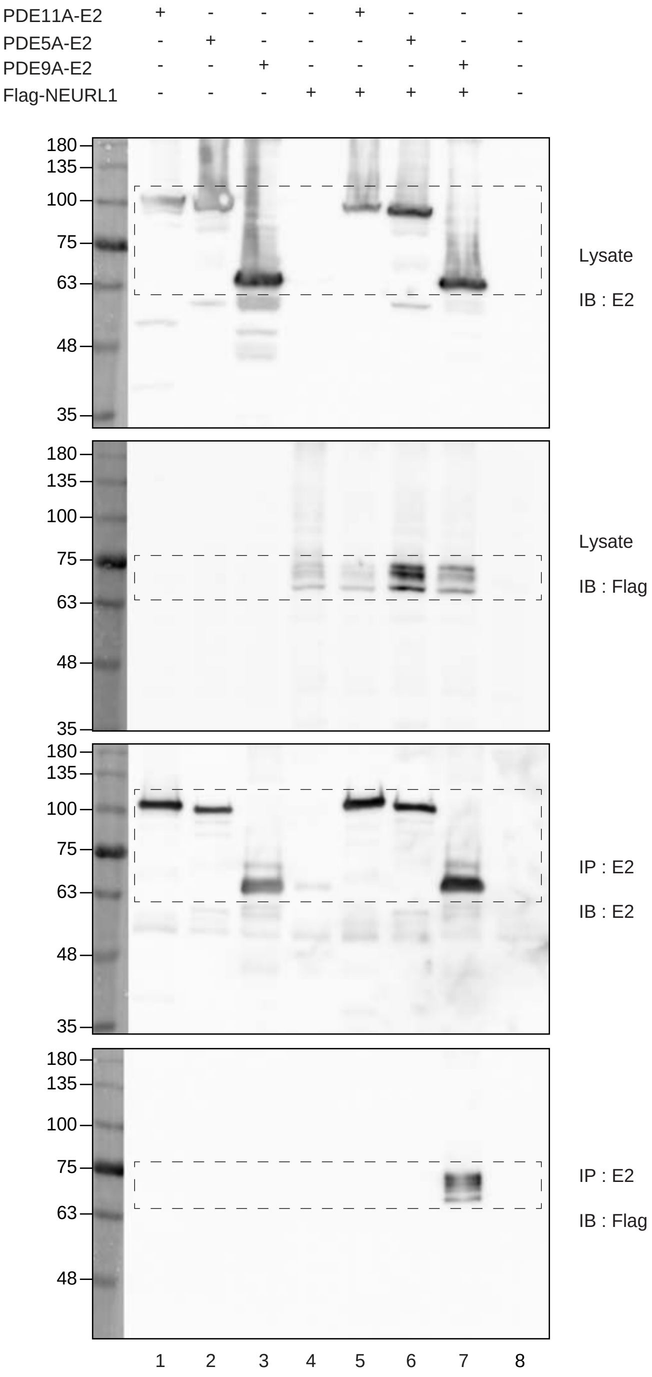

PDE11A-E2 + - - - + - - -
PDE5A-E2 - + - - - + - -
PDE9A-E2 - - + - - - + -
Flag-NEURL1 - - - + + + + -
180
135
100
75
63
48
35
Lysate
IB : E2
Lysate
IB : Flag
IP : E2
IB : E2
IP : E2
IB : Flag
1 2 3 4 5 6 7 8

# Figure 2B

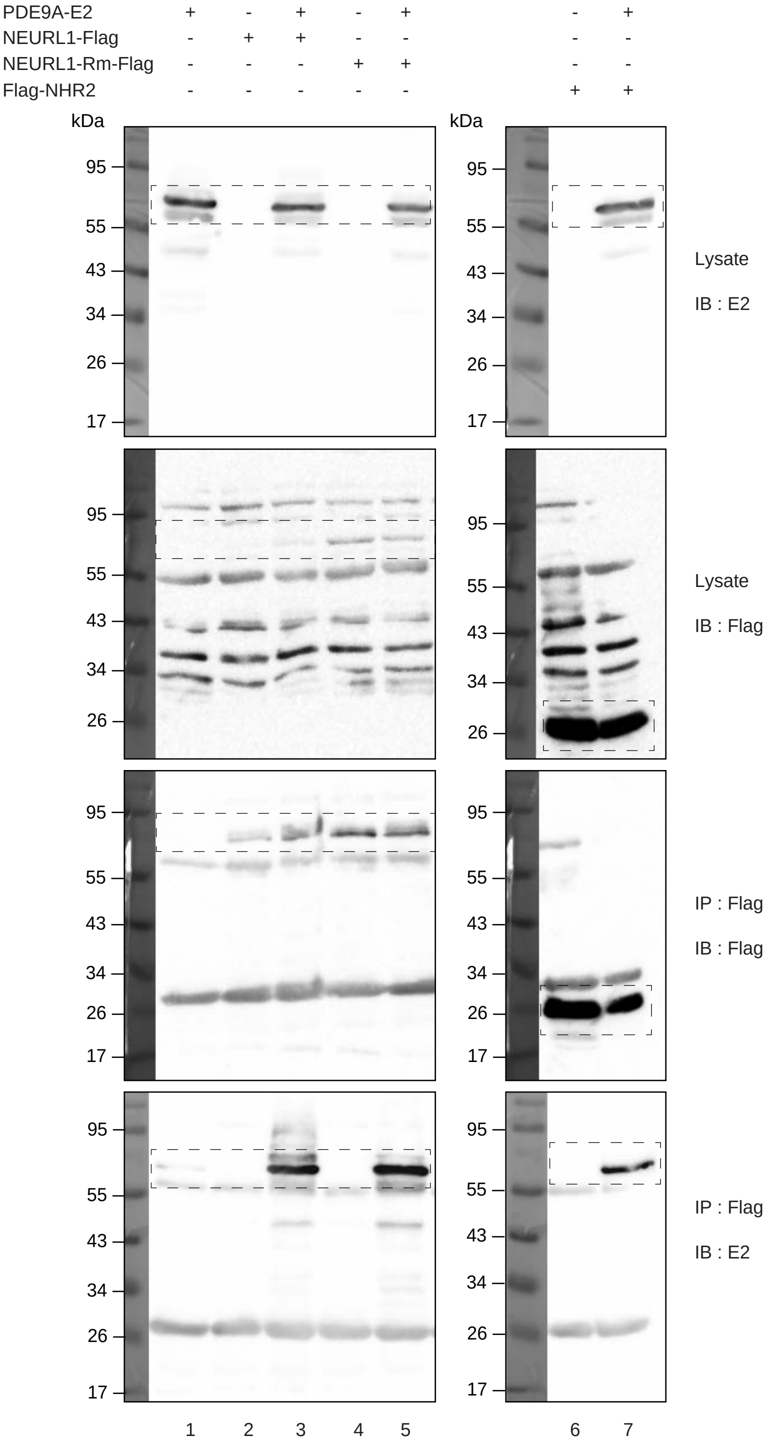
PDE9A-E2 + - + - + - +
NEURL1-Flag - + + - - - -
NEURL1-Rm-Flag - - - + + - -
Flag-NHR2 - - - - - + +
kDa
95
55
43
34
26
17
Lysate
IB : E2
Lysate
IB : Flag
IP : Flag
IB : Flag
IP : Flag
IB : E2
1 2 3 4 5 6 7

# Figure 2C

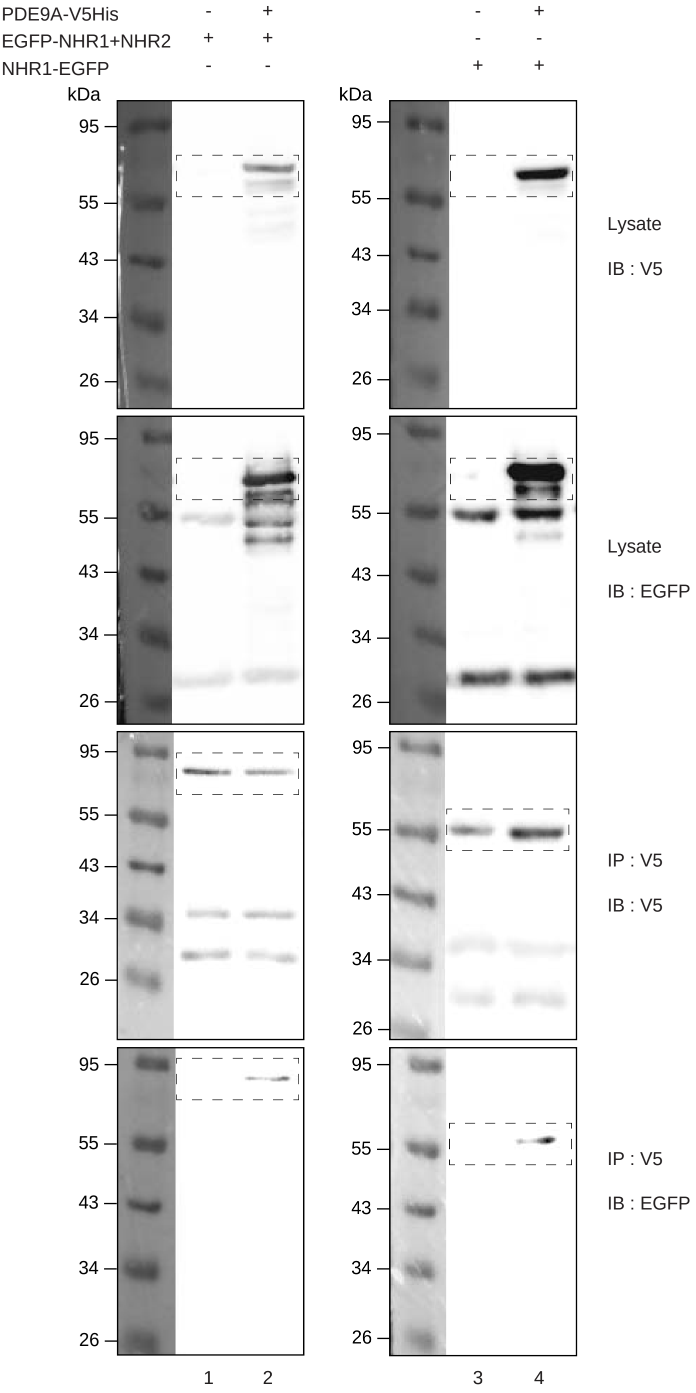

PDE9A-V5His - + - +
EGFP-NHR1+NHR2 + + - -
NHR1-EGFP - - + +
kDa
95
55
43
34
26
Lysate
IB : V5
Lysate
IB : EGFP
IP : V5
IB : V5
IP : V5
IB : EGFP
1 2 3 4

# Figure 2D

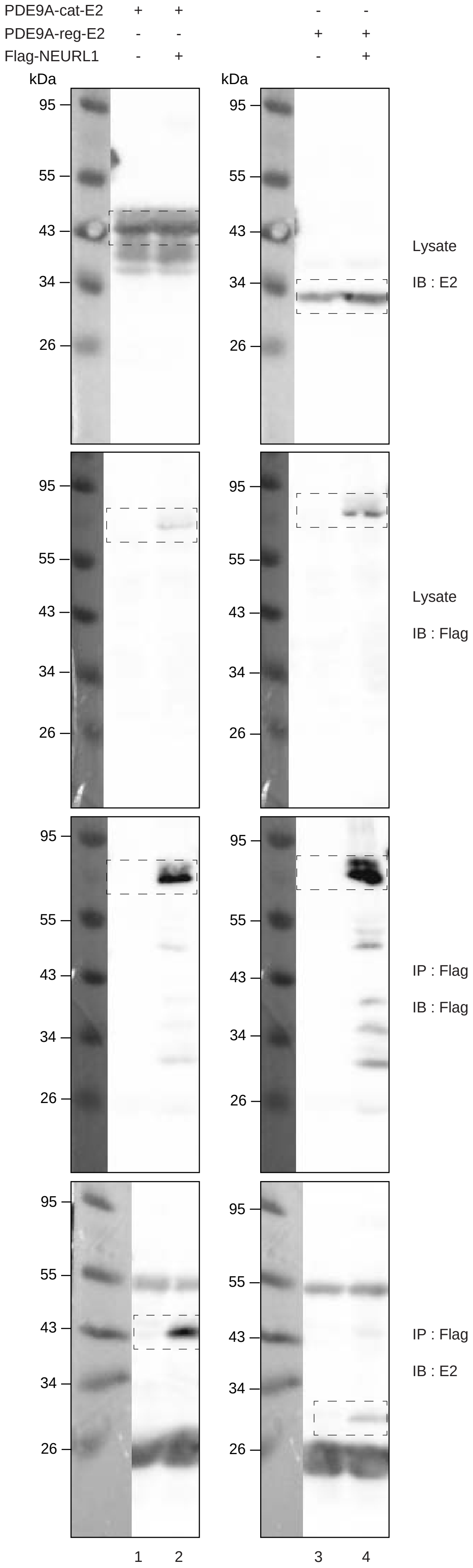
PDE9A-cat-E2 + + - -
PDE9A-reg-E2 - - + +
Flag-NEURL1 - + - +
kDa
95
55
43
34
26
Lysate
IB : E2
Lysate
IB : Flag
IP : Flag
IB : Flag
IP : Flag
IB : E2
1 2 3 4

# Figure 3A

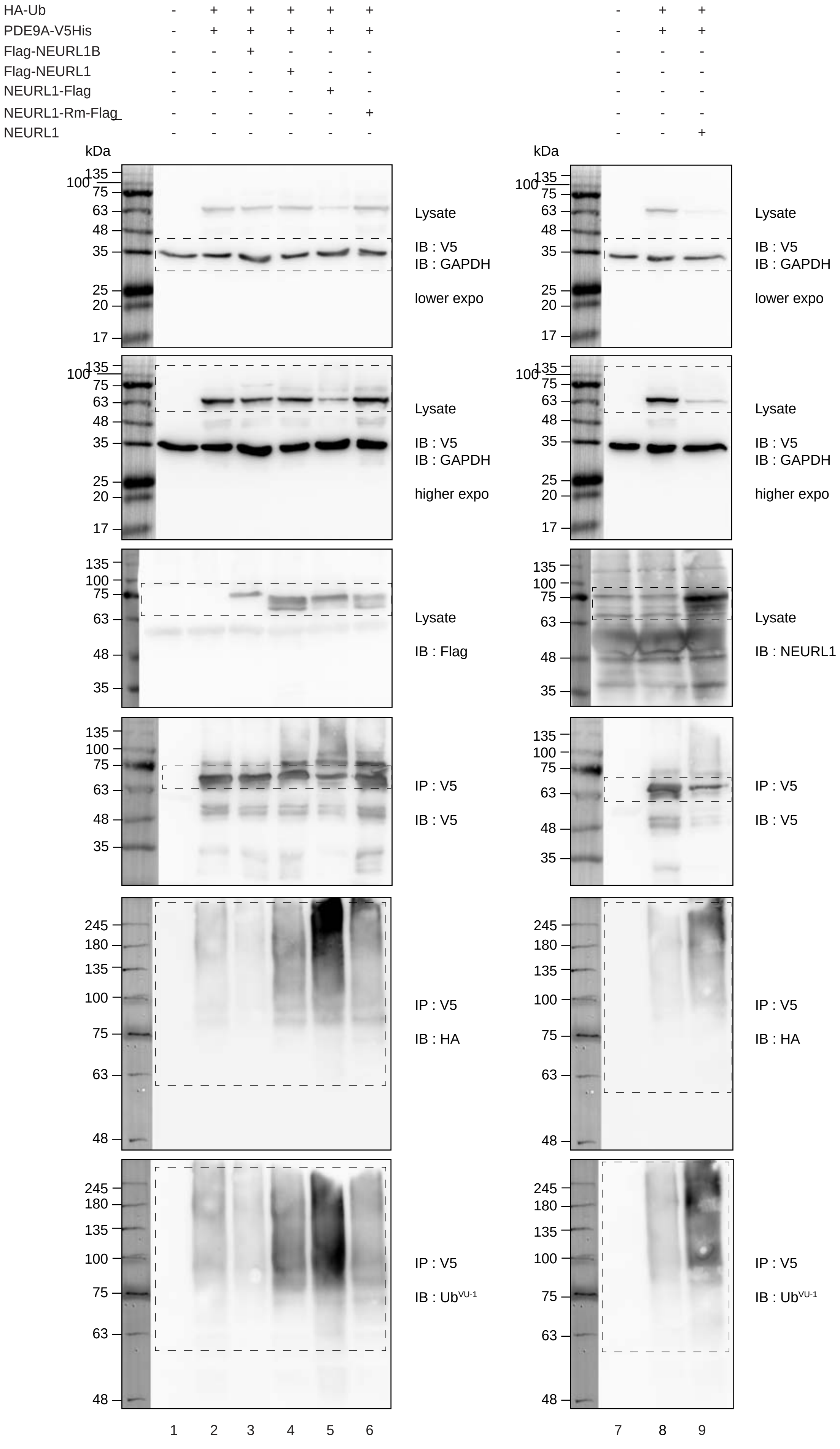
HA-Ub - + + + + + - + +
PDE9A-V5His - + + + + + - + +
Flag-NEURL1B - - + - - - - - -
Flag-NEURL1 - - - + - - - - -
NEURL1-Flag - - - - + - - - -
NEURL1-Rm-Flag - - - - - + - - -
NEURL1 - - - - - - - - +
kDa
135
100
75
63
48
35
25
20
17
Lysate
IB : V5
IB : GAPDH
lower expo
higher expo
IB : Flag
IB : NEURL1
IP : V5
245
180
IB : HA
IB : Ub VU-1
1 2 3 4 5 6 7 8 9

# Figure 3B

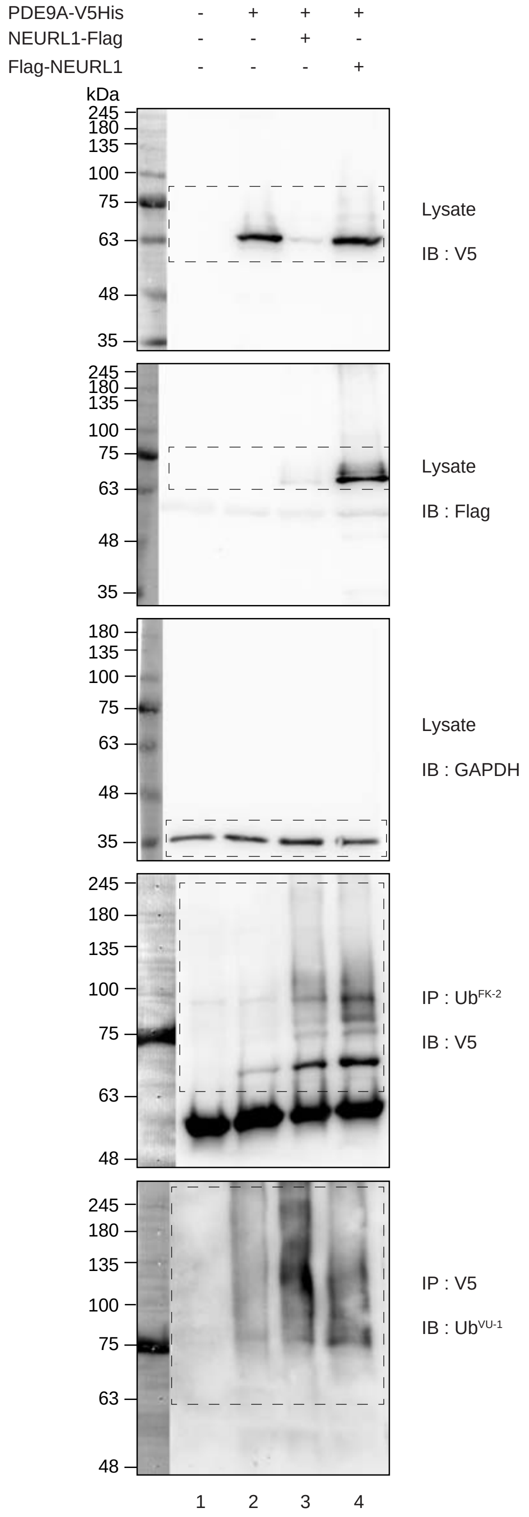

PDE9A-V5His - + + +
NEURL1-Flag - - + -
Flag-NEURL1 - - - +
kDa
245
180
135
100
75
63
48
35
Lysate
IB : V5
Lysate
IB : Flag
Lysate
IB : GAPDH
IP : Ub^FK-2
IB : V5
IP : V5
IB : Ub^VU-1
1 2 3 4

# Figure 4A

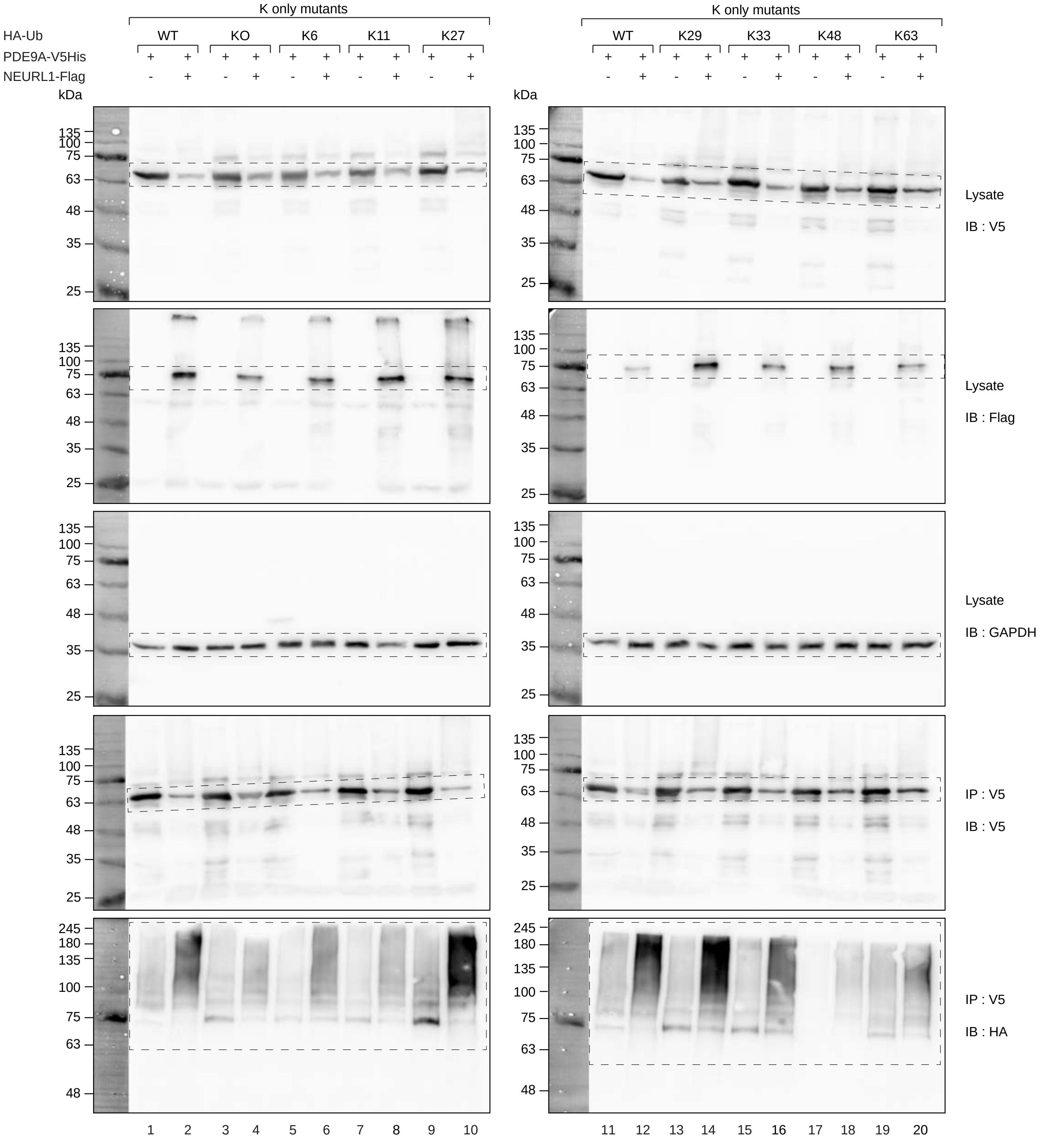
K only mutants
K only mutants
HA-Ub
WT
KO
K6
K11
K27
WT
K29
K33
K48
K63
PDE9A-V5His
NEURL1-Flag
kDa
135
100
75
63
48
35
25
245
180
Lysate
IB : V5
Lysate
IB : Flag
Lysate
IB : GAPDH
IP : V5
IB : V5
IP : V5
IB : HA
1
2
3
4
5
6
7
8
9
10
11
12
13
14
15
16
17
18
19
20

# Figure 4B

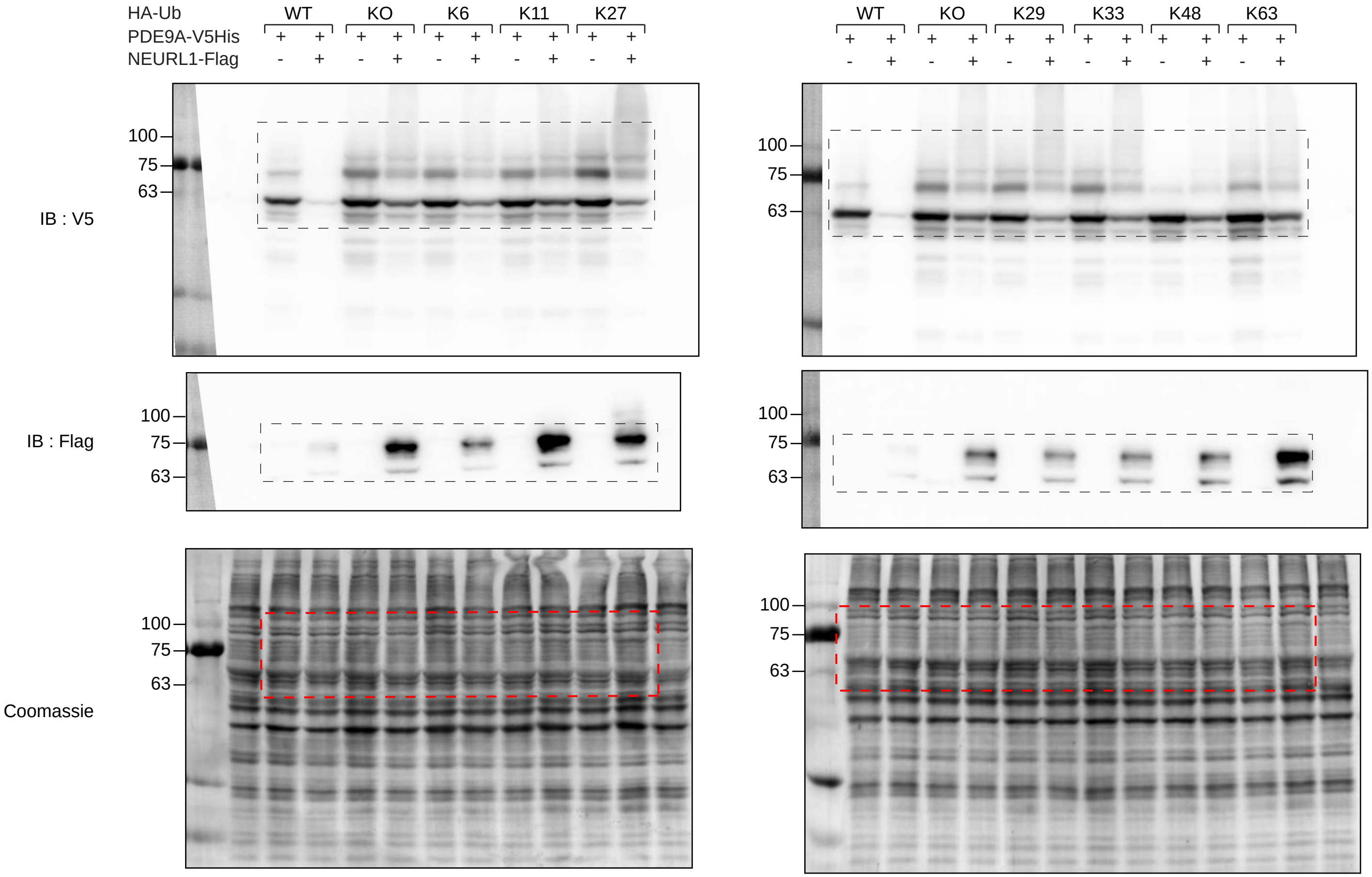

HA-Ub
PDE9A-V5His
NEURL1-Flag
WT
KO
K6
K11
K27
+ + + + + + + + + +
- + - + - + - + - +
WT
KO
K29
K33
K48
K63
+ + + + + + + + + + + +
- + - + - + - + - + - +
IB : V5
100
75
63
IB : Flag
100
75
63
Coomassie
100
75
63

# Figure 5A

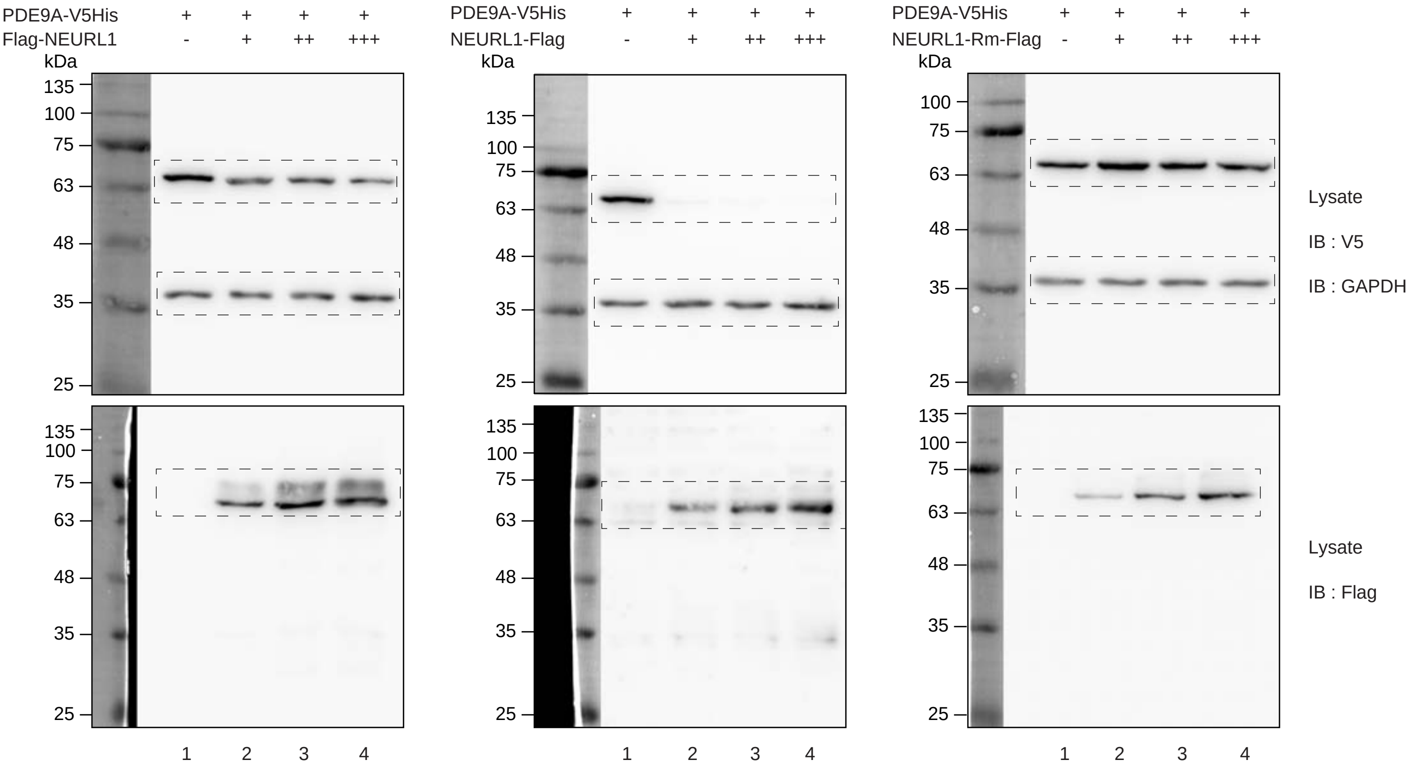
PDE9A-V5His + + + +
Flag-NEURL1 - + ++ +++
kDa
135
100
75
63
48
35
25
PDE9A-V5His + + + +
NEURL1-Flag - + ++ +++
kDa
PDE9A-V5His + + + +
NEURL1-Rm-Flag - + ++ +++
kDa
Lysate
IB : V5
IB : GAPDH
Lysate
IB : Flag
1 2 3 4
1 2 3 4
1 2 3 4

# Figure 5B

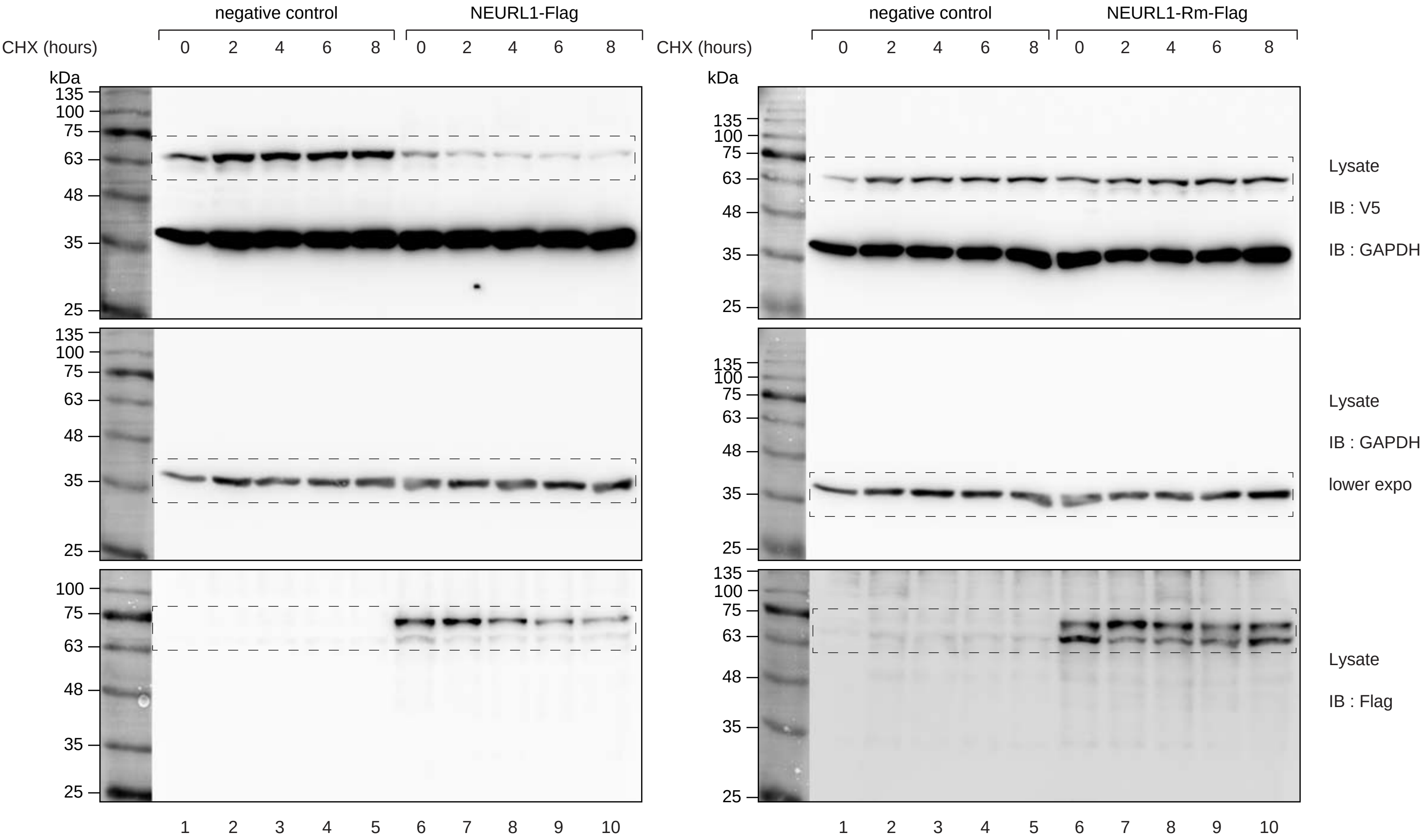
negative control
NEURL1-Flag
CHX (hours)
0 2 4 6 8 0 2 4 6 8
negative control
NEURL1-Rm-Flag
CHX (hours)
0 2 4 6 8 0 2 4 6 8
kDa
135
100
75
63
48
35
25
Lysate
IB : V5
IB : GAPDH
Lysate
IB : GAPDH
lower expo
Lysate
IB : Flag
1 2 3 4 5 6 7 8 9 10

# Figure 5C

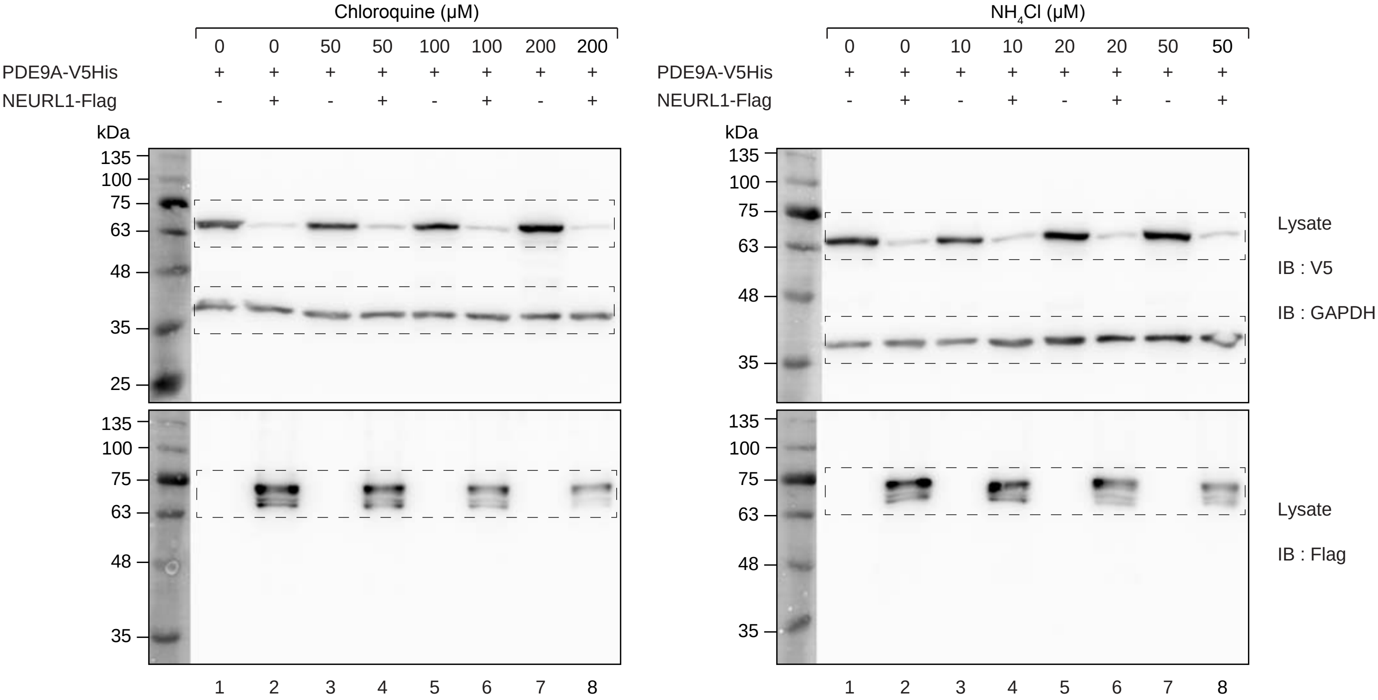

Chloroquine (μM)
0 0 50 50 100 100 200 200
PDE9A-V5His + + + + + + + +
NEURL1-Flag - + - + - + - +
kDa
135
100
75
63
48
35
25
1 2 3 4 5 6 7 8
NH4Cl (μM)
0 0 10 10 20 20 50 50
PDE9A-V5His + + + + + + + +
NEURL1-Flag - + - + - + - +
Lysate
IB : V5
IB : GAPDH
Lysate
IB : Flag

# Figure 5D

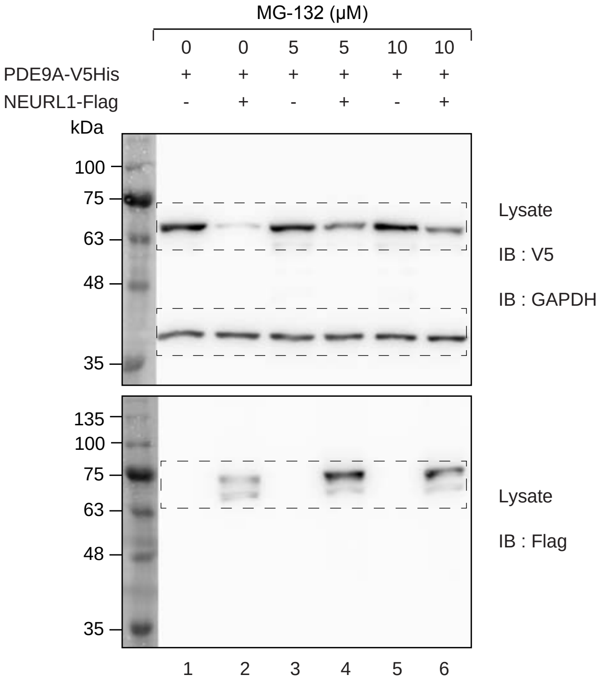

MG-132 (μM)
0 0 5 5 10 10
PDE9A-V5His + + + + + +
NEURL1-Flag - + - + - +
kDa
100
75
63
48
35
Lysate
IB : V5
IB : GAPDH
135
100
75
63
48
35
Lysate
IB : Flag
1 2 3 4 5 6

# Figure 6

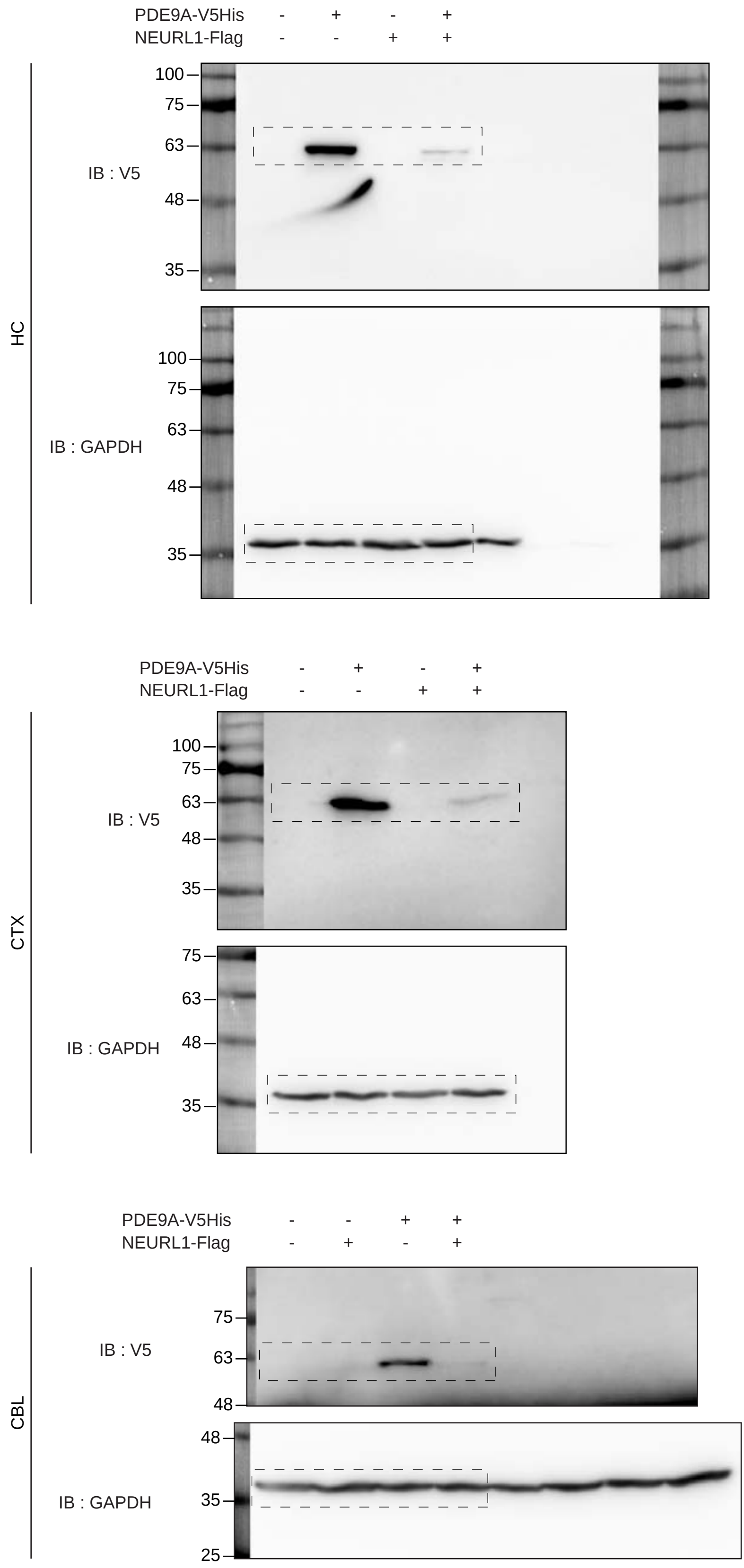

PDE9A-V5His - + - +
NEURL1-Flag - - + +
HC
IB : V5
100
75
63
48
35
IB : GAPDH
100
75
63
48
35
PDE9A-V5His - + - +
NEURL1-Flag - - + +
CTX
IB : V5
100
75
63
48
35
IB : GAPDH
75
63
48
35
PDE9A-V5His - - + +
NEURL1-Flag - + - +
CBL
IB : V5
75
63
48
IB : GAPDH
48
35
25
